# Life-Course Persistent Antisocial Behavior and Accelerated Biological Aging in a Longitudinal Birth Cohort

**DOI:** 10.3390/ijerph192114402

**Published:** 2022-11-03

**Authors:** Stephanie Langevin, Ashalom Caspi, J. C. Barnes, Grace Brennan, Richie Poulton, Suzanne C. Purdy, Sandhya Ramrakha, Peter T. Tanksley, Peter R. Thorne, Graham Wilson, Terrie E. Moffitt

**Affiliations:** 1 Department of Psychology and Neuroscience, Duke University, Durham, NC 27708, USA; 2 Department of Psychiatry and Behavioral Sciences, Duke University, Durham, NC 27708, USA; 3 Center for Genomic and Computational Biology, Duke University, Durham, NC 27708, USA; 4 Geriatric Research, Education, and Clinical Center, Durham VA Healthcare System, Durham, NC 27705, USA; 5 School of Criminal Justice, University of Cincinnati, Cincinnati, OH 45221, USA; 6 Department of Psychology, University of Otago, Dunedin 9016, New Zealand; 7 Discipline of Speech Science, University of Auckland, Auckland 1142, New Zealand; 8 Population Research Center, University of Texas at Austin, Austin, TX 78705, USA; 9 Discipline of Audiology, University of Auckland, Auckland 1142, New Zealand; 10 Matai Medical Research Institute, Gisborne 4010, New Zealand

**Keywords:** antisocial trajectories, biological aging, crime, accelerated aging

## Abstract

Prior research shows that individuals who have exhibited antisocial behavior are in poorer health than their same-aged peers. A major driver of poor health is aging itself, yet research has not investigated relationships between offending trajectories and biological aging. We tested the hypothesis that individuals following a life-course persistent (LCP) antisocial trajectory show accelerated aging in midlife. Trajectories of antisocial behavior from age 7 to 26 years were studied in the Dunedin Multidisciplinary Health and Development Study, a population-representative birth cohort (N = 1037). Signs of aging were assessed at age 45 years using previously validated measures including biomarkers, clinical tests, and self-reports. First, we tested whether the association between antisocial behavior trajectories and midlife signs of faster aging represented a decline from initial childhood health. We then tested whether decline was attributable to tobacco smoking, antipsychotic medication use, debilitating illnesses in adulthood, adverse exposures in childhood (maltreatment, socioeconomic disadvantage) and adulthood (incarceration), and to childhood self-control difficulties. Study members with a history of antisocial behavior had a significantly faster pace of biological aging by midlife, and this was most evident among individuals following the LCP trajectory (β, 0.22, 95%CI, 0.14, 0.28, *p* ≤ 0.001). This amounted to 4.3 extra years of biological aging between ages 25–45 years for Study members following the LCP trajectory compared to low-antisocial trajectory individuals. LCP offenders also experienced more midlife difficulties with hearing (β, −0.14, 95%CI, −0.21, −0.08, *p* ≤ 0.001), balance (β, −0.13, 95%CI, −0.18, −0.06, *p* ≤ 0.001), gait speed (β, −0.18, 95%CI, −0.24, −0.10, *p* ≤ 0.001), and cognitive functioning (β, −0.25, 95%CI, −0.31, −0.18, *p* ≤ 0.001). Associations represented a decline from childhood health. Associations persisted after controlling individually for tobacco smoking, antipsychotic medication use, midlife illnesses, maltreatment, socioeconomic status, incarceration, and childhood self-control difficulties. However, the cumulative effect of these lifestyle characteristics together explained why LCP offenders have a faster Pace of Aging than their peers. While older adults typically age-out of crime, LCP offenders will likely age-into the healthcare system earlier than their chronologically same-aged peers. Preventing young people from offending is likely to have substantial benefits for health, and people engaging in a LCP trajectory of antisocial behaviors might be the most in need of health promotion programs. We offer prevention and intervention strategies to reduce the financial burden of offenders on healthcare systems and improve their wellbeing.

## 1. Introduction

Antisocial behavior is the providence of youth. The age-crime curve documents that the onset of antisocial behavior is typically between ages 8 and 14 years, and it peaks between ages 15 and 19 years, followed by a gradual desistance period, typically between ages 20 and 29 years [1,2]. However, what happens to antisocial youth as they age? Research suggests that individuals who exhibited conduct problems in their youth utilize high levels of healthcare services as adults [3]. Similarly, individuals exhibiting antisocial behaviors were found to be in poorer health than their peers in adulthood. Thus, we can expect an increase in service usage and disease burden among these high-need/high-cost antisocial individuals [3,4]. Here, we present a test of the hypothesis that people exhibiting antisocial behaviors age faster than their peers; that is, they experience accelerated physiological decline toward age-related disease and mortality. We tested this hypothesis in a population-representative cohort of New Zealanders who have been followed from birth to midlife. This longitudinal study has two goals. First, in relation to theory, we aim to bridge insights about developmental criminology with the emerging field of geroscience. Second, in relation to practice and public health, we aim to shed light on the link between crime and physical health across the life span.

Offenders are significantly more likely to die prematurely compared to non-offenders [5,6], a statistic only partly explained by unnatural causes of death, such as homicide and suicide. A recent meta-analysis of 36 studies (N = 1,116,614) on crime and health showed that people who offend are over three times more likely to die prematurely, and this mortality gap is in part attributable to deaths from physical disease. Indeed, deaths from physical disease are twice as likely among offenders compared with non-offenders [7]. Having a history of antisocial behavior is associated with multiple age-related conditions and diseases, including hypertension, diabetes, arthritis, cardiovascular diseases, respiratory diseases and cancer [6,8]. A major driver of such medical conditions is the process of aging itself [9,10]. Biological aging is the gradual, progressive coordinated decline in integrity across multiple organ systems that occurs with advancing chronological age, causing morbidity and mortality [11]. While all individuals age chronologically at the same rate, there is marked variation in their rate of biological aging, some adults experience age-related decline faster than others [12,13]. Yet, few studies have investigated biological aging among a segment of the population especially at risk for faster biological aging and earlier onset of age-related disease: those who engage in antisocial behaviors. We tested the hypothesis that accelerated biological aging may accompany antisocial behavior, which tends to onset and peak in the first two decades, because this might lead to physical disease, which onset and peak decades later [14].

To test the association between a history of antisocial behavior and accelerated aging, we turned to the developmental taxonomy of antisocial behavior which proposes that the age-crime curve conceals distinct groups of antisocial individuals [15]. The theory distinguishes between life-course persistent versus adolescence-limited antisocial behavior trajectories (hereafter LCP and AL). LCP individuals’ antisocial behavior has its origins in neurodevelopmental processes and family adversity, beginning in childhood, and building persistently to construct a disordered personality with hallmark features of physical aggression. In contrast, AL’s antisocial activities have their origins in age-graded social processes that begin with a maturity gap in adolescence and end when social adulthood is attained. According to the taxonomy, LCP antisocial behavior is rare, persistent, pervasive and pathological, whereas AL antisocial behavior is common, relatively transient, situational and near normative. Subsequent research has revealed a third trajectory of antisocial behavior characterized by childhood-limited (CL) conduct problems which desist before adolescence [16]. Developmental research has confirmed the distinct etiology of these antisocial trajectories [17]. A recent investigation of the link between criminal career and early death among a 1950s cohort of English delinquents found limited evidence for an association between LCP offenders, career duration and early death [18]. These results were attributable, at least in part, to truncation by unnatural death (e.g., homicide, suicide), as suggested by the authors. However, based on evidence that the mortality gap between offenders and non-offenders is only partially explained by unnatural causes of death, we sought to investigate whether different developmental trajectories of antisocial behavior are associated with accelerated pace of aging (i.e., a potential factor in early mortality) up to midlife in a 1970s birth cohort.

To capture signs of aging, we evaluated the coordinated, progressive loss of integrity across cardiovascular, metabolic, pulmonary, kidney, immune, and dental systems using a 19-biomarker composite measure of the pace of aging that has previously been developed and validated [13]. This longitudinal measure captures correlated gradual decline across organ systems from age 26 to 45 years and reflects differences among individuals in their rate of biological aging. Accelerated pace of aging is known to predict elevated risk of disease multimorbidity, including dementia, and early mortality [19,20,21]. In addition to the pace of aging, we also measured sensory, motor, and cognitive functioning at midlife using assessments that are typically used in gerontology. We also measured observer ratings of participants’ facial age at midlife. Finally, to provide a proof-of-principle that replications and extensions of this study are possible for research teams that do not have two decades of repeated health measures, we also tested a DNA methylation biomarker measure of pace of aging called DunedinPACE (for **P**ace of **A**ging **C**alculated from the **E**pigenome). It distills our 19-indicator longitudinal measure of the decline in organ-system integrity into an easy-to implement, single-time-point DNA-methylation blood-test.

Antisocial individuals who experience accelerated aging might also have been in worse health as children [10]. As such, we tested whether the link between different antisocial behavior trajectories and indicators of aging captured a decline from initial childhood health. In addition, antisocial individuals may also be in otherwise poorer health as adults. Thus, we tested whether the link between antisocial behavior and accelerated aging was attributable to tobacco smoking, the use of antipsychotic medication whose side effects mimic signs of accelerated aging [22], or to debilitating illnesses in adulthood. There is also a need to rule out confounding by adverse childhood (e.g., maltreatment or growing up in poverty) and adult (e.g., incarceration) exposures that may lead to accelerated aging [23,24,25,26,27,28,29]. We then tested whether the association between antisocial behavior trajectories and accelerated aging could be explained by childhood self-control difficulties that set in motion both lifelong antisocial behavior and accelerated aging [26]. Finally, we tested whether individuals exhibiting increased antisocial behaviors are also those who have the least health literacy, potentially hindering future health promoting behaviors.

The overarching aim of this study was to investigate the association between antisocial behavior trajectories and signs of accelerated aging by midlife in a prospective, population-based cohort. Four specific aims were investigated. First, we investigated whether the three antisocial trajectories (life-course persistent, childhood-limited, adolescence-limited) were associated with midlife indicators of aging. Second, we tested whether this association captured a decline from initial childhood health. Third, we investigated the contribution of environmental and individual-level risk exposures in accounting for the association between different antisocial behavior trajectories and signs of accelerated aging. Finally, we investigated group differences in health knowledge.

## 2. Methods

### 2.1. Participants

Participants were members of the Dunedin Multidisciplinary Health and Development Study, a longitudinal investigation of health and behavior in a complete birth cohort. Dunedin participants (n = 1037, 91% of eligible births, 52% male) were all individuals born between April 1972 and March 1973 in Dunedin, New Zealand, who were eligible based on residence in the province at age 3 years and who participated in the first assessment at age 3 years. Details are reported elsewhere [30]. The cohort represented the full range of socioeconomic status in the general population of New Zealand’s South Island. The cohort matches the New Zealand National Health and Nutrition Survey on key health indicators (e.g., body mass index, smoking, physical activity, and visits to the doctor) and matches the New Zealand Census of people the same age on educational attainment.

Assessments were conducted at birth, at ages 3, 5, 7, 9, 11, 13, 15, 18, 21, 26, 32, and 38 years and, most recently, at age 45 years when 938 of the 997 participants (94.1%) still alive participated. Participants who took part in assessments at age 45 years did not differ significantly from other living participants in terms of childhood social class, childhood IQ, or childhood self-control (Supplementary Materials, Attrition analyses).

At each assessment, participants are brought to the research unit for interviews and examinations. These data are supplemented by searches of official records and by questionnaires that are mailed, as developmentally appropriate, to parents, teachers, and peers nominated by the participants themselves. The Dunedin Study was approved by the NZ-HDEC (Health and Disability Ethics Committee). Study members gave written informed consent before participating.

### 2.2. Assessment of Antisocial Behavior Trajectories

As previously described [16], Study members were classified as following a life-course-persistent (LCP), adolescence-limited (AL), childhood-limited (CL) or low antisocial behavior trajectory, on the basis of growth-mixture modelling applied to six facets of antisocial behavior assessed at ages 7, 9, 11, 13, 15, 18, 21, and 26: physical fighting, bullying, destroying property, lying, stealing, and truancy (or chronic work absenteeism). The LCP antisocial behavior group met conduct disorder diagnostic criteria at ages 7, 9, 11, 13 and 15, and the AL antisocial behavior group met criteria at age 15 [16]. After age 26, the antisocial behavior groups continued to diverge in antisocial behavior; according to nationwide records, between ages 26–38, LCP individuals were convicted 5.3 times on average, the AL group 1.1 times, and the low-antisocial group 0.31 times [17]. By age 45 years, 2.9% (n = 15) of individuals in the low antisocial trajectory group had died, compared to 2.6% (n = 6) of the CL group, 6.3% (n = 12) of the AL group, and 4.3% (n = 4) of the LCP antisocial group.

Of the 938 Study members assessed at age 45, 934 had been classified into an antisocial group and could be included in the analyses: 8.9% (n = 83) were identified as following a LCP antisocial behavior trajectory, 22.2% (n = 207) a CL antisocial behavior trajectory, 18.5% (n = 173) an AL antisocial behavior trajectory, and 50.4% (n = 471) a low level of antisocial behavior trajectory, totaling 934 participants. The four participants with missing antisocial behavior trajectory data were excluded from this study.

### 2.3. Measuring Signs of Accelerated Aging in Midlife

We measured Study members’ pace of biological aging, plus the integrity of sensory and motor systems (hearing, balance, motor functioning, and vision), and cognitive functioning at age 45 years by using previously validated measures based on assessments typical of gerontology (Supplementary Materials).

*Pace of Aging* was measured for each Study member with repeated assessments of a panel of 19 biomarkers taken at ages 26, 32, 38, and 45 years, a method previously described [13]. The 19 biomarkers were as follows: body mass index, waist–hip ratio, glycated hemoglobin (HbA1C), leptin, blood pressure (mean arterial pressure), cardiorespiratory fitness (VO2Max), forced expiratory volume in one second (FEV1), forced vital capacity ratio (FEV1/FVC), total cholesterol, triglycerides, high-density lipoprotein cholesterol, apolipoprotein B100/A1 ratio, lipoprotein(a), creatinine clearance, urea nitrogen, C-reactive protein, white blood cell count, gum health, and caries-affected tooth surfaces. We modeled change over time in each biomarker with 19 mixed-effects growth models and composited the results (scaled by sex) within each individual to calculate their Pace of Aging as years of physiological change occurring per one chronological year.

*Social hearing* (the ability to hear in noisy environments) was measured using the Listening in Spatialized Noise-Sentences Test (LiSN-S). The test determines speech reception thresholds (dB) for sentences presented in competing speech under various conditions that spatially separate the test sentences from competing speech. For our analyses, we used a measure of low cue speech reception threshold, reflecting hearing performance when the person is not receiving optimum auditory information [31]. Lower score indicates worse performance.

*Balance* was measured using the Unipedal Stance Test, as the maximum time achieved standing on one leg across 3 trials of the test with eyes closed [32]. A lower score indicates worse performance.

*Gait speed* (m/s) was measured using the GAITRite Electronic Walkway (CIR Systems Inc). Gait speed was assessed under 3 conditions: usual gait speed (walk at normal pace; mean of 2 walks) and 2 challenging paradigms: dual task gait speed (walk at normal pace while reciting alternate letters of the alphabet out loud; mean of 2 walks), and maximum gait speed (walk as fast as safely possible; mean of 3 walks). We calculated the mean of the 3 individual walk conditions to generate a reliable measure of composite gait speed [33]. A lower score indicates slower gait speed.

*Visual Contrast sensitivity* was measured using a Thomson Test Chart administered by trained technicians. The chart presents 3 letters per line and the letters gradually fade from black to grey to white on a white background to determine to lowest level of “contrast” that the eye can detect. The resulting measure is a contrast sensitivity score function (CSF), reflecting a person’s best-corrected contrast-detection threshold, the lowest contrast at which a pattern can be seen. Lower scores indicate worse visual contrast sensitivity capability at age 45.

*Cognitive ability* was measured using the Wechsler Adult Intelligence Scale-IV (WAIS-IV) [34]. Lower scores represent lower cognitive ability.

*DunedinPACE* was previously derived to develop a DNA methylation algorithm to predict the 20-year Pace of Aging measure using Illumina EPIC array DNA methylation data from blood collected at age 45. The resulting algorithm included 173 CpG sites and demonstrated a high in-sample correlation with Pace of Aging at age 45 [19]. Results are modelled as years of physiological change occurring per one chronological year.

*Facial age* was measured by an independent panel of eight raters of each participant’s digital neutral facial photograph and coded such that higher scores indicate older appearance.

### 2.4. Measuring Childhood Health Indicators

We matched each of the midlife aging outcomes to prospectively collected childhood measures (e.g., we matched adult gait speed with childhood motor development) in order to control for baseline childhood differences most relevant to each midlife outcome assessed (Supplementary Materials). This enabled us to test whether associations between antisocial behavior and midlife outcomes represent a relative decline from childhood health. Table 1 shows the childhood health indicators in each of the four antisocial behavior trajectory groups.

*Childhood physical health* between birth and age 11 was measured using birth records, medical exams, anthropometry, lung function testing, nurse ratings, and clinical interviews with parents about health conditions (e.g., asthma, childhood diabetes) [35].

*Childhood social hearing* at age 11 was assessed using a speech-in-noise test (SPIN). The test measures children’s ability to correctly identify words under conditions of no noise, and +10 and +5 dB SPIN ratios. To approximate the childhood measure to the adult social hearing measure, we used children’s performance in the +5 dB SPIN ratio condition, reflecting social hearing ability in the most difficult auditory environment [36].

*Childhood balance* at ages 3, 7, and 9 years was measured using the balance subtests of the Bayley Motor Scales (age 3) and of the Basic Motor Ability Test (ages 7 and 9) [37,38].

*Childhood vision* at ages 7, 9 and 11 years was measured using the Sheridan Gardiner single optotype letter matching test at 6m (age 7) and a 4-m logarithmic test chart (ages 9 and 11).

*Childhood motor development* at ages 3, 5, 7 and 9 years was measured using the Bayley Motor Scales (age 3) and the Basic Motor Ability Test (ages 7 and 9) [37,38]. To construct a cross-age measure of childhood motor development, assessments were standardized to M = 0 SD = 1 within age, and then averaged across ages.

*Childhood cognitive ability* was measured using the Wechsler Intelligence Scale for Children-Revised (WISC-R), individually administered at ages 7, 9, and 11 years. Scores for the 3 ages were averaged [39].

### 2.5. Measuring Health Issues in Adulthood

*Tobacco smoking* was coded as whether participants had reported daily tobacco smoking at any assessment up to age 45 years (478 participants [51.6% of cohort members]). *Antipsychotic medication use* at age 45 years was assessed in standardized interviews about medications, and participants brought medications on the assessment day, which were evaluated by a pharmacist. Antipsychotics were used by 18 participants (1.9% of cohort members). *Cancer* or *heart attack* by age 45 years was assessed by standardized interviews that ascertained whether participants had been told by a health professional that they had these diseases. *Diabetes* was assessed based on participants’ blood levels of glycated hemoglobin. In line with clinical diagnostic criteria, a cutoff of 48 mmol/mol was used. Cancer, heart attack, or diabetes by age 45 years affected 56 participants (6.2% of cohort members). Table 1 shows the prevalence of these health conditions in each of the four antisocial behavior trajectory groups for the participants of this study.

### 2.6. Measuring Adverse Experiences

*Childhood socioeconomic status* of participants’ families was measured using the 6-point Elley-Irving socioeconomic index for New Zealand that assessed parents’ occupational statuses, defined based on average income and educational levels derived from the New Zealand Census [40,41]. *Childhood maltreatment* includes evidence of (1) maternal rejection assessed at age 3 years by observational ratings of mothers’ interaction with the study children, (2) harsh discipline assessed at ages 7 and 9 years by parental report of disciplinary behaviours, (3) two or more changes in the child’s primary caregiver, and (4) physical abuse, and (5) sexual abuse reported by study members once they reached adulthood. For each child, our cumulative index counts the number of maltreatment indicators during the first decade of life; 64.2% of the full cohort experienced no maltreatment, 26.6% experienced 1 indicator of maltreatment (“probable” maltreatment), and 9.2% experienced 2 or more indicators of maltreatment (“definite” maltreatment) [42]. *Lifetime incarceration* was assessed via self-report at ages 32, 38 and 45 years. At age 32, participants were asked, “In your life, have you ever spent any time in jail or prison?”. At phase 38 and 45, this was updated with months of incarceration between phases. Reports were combined to create lifetime months of incarceration. A total of 39 participants (4.1% of the full cohort) were identified as having spent at least one month in jail or prison. Table 1 shows the distribution of these environmental experiences among each of the four antisocial behavior trajectory groups for the participants of this study.

### 2.7. Measuring Early Childhood Self-Control Difficulties

*Children’s self-control difficulties* were measured at ages 3 and 5 years, before the assessment of antisocial behavior trajectories. At ages 3 and 5 years, each child participated in cognitive and motor tasks. The children were tested by examiners who had no knowledge of their behavioral history, and who rated the child’s lack of control after the testing session [43,44]. Table 1 shows the mean levels of self-control difficulties among each of the four antisocial behavior trajectory groups for the participants of this study.

### 2.8. Measuring Health Knowledge

To ascertain study members’ *health knowledge* at age 45 we administered two measures. First, a multiple-choice scale included 6 items related to medical knowledge, prevention, aging, physical disease, sun exposure, and sleep. Second, in an open-ended interview, participants explained their understanding of different health principles such as: “What are some of the reasons you should know your family history of illness?”; “If you are sick and the doctor gives you an antibiotic, what are some of the reasons why you should finish all the pills?”; “What are some of the reasons people tend to gain weight as they get older?”. Using standardized scoring procedures, a panel of two raters coded responses on a scale from 0 to 2. The Practical Health Knowledge measure was computed by standardizing (M = 0, SD = 1) and averaging the multiple-choice and open-ended scales [26].

### 2.9. Statistical Analysis

Analyses were conducted in seven steps. First, regression analyses were conducted to test the association between antisocial behavior trajectories and signs of accelerated aging in midlife. In all analyses we controlled for sex and used dummy coding for the antisocial trajectories, with the low trajectory of antisocial behavior as the reference category. Second, we added, to each regression model, a prospectively-collected childhood health indicator matched to each of the midlife outcomes to test whether associations between antisocial behavior trajectories and midlife outcomes represent a decline from childhood health. Third, we successively included indicators of poor adult health that may accelerate aging: tobacco smoking, antipsychotic medication use, and cancer, heart attack, or diabetes. Fourth, we successively added information about adverse experiences that might accelerate aging (childhood SES, childhood maltreatment, incarceration). Fifth, we tested whether antisocial behavior trajectories remained associated with signs of accelerated aging when accounting for early childhood self-control difficulties, an individual characteristic known to predict both antisocial behavior and accelerated aging. Sixth, we entered all covariates together, to test if accumulating background and lifestyle factors could explain associations between antisocial trajectories and aging outcomes. Finally, we looked at group differences in health knowledge, which could affect their future (un)healthy behaviors and aging. We present standardized regression coefficients (β) for all associations. Statistical analyses were performed in Mplus, version 7.2 using full information maximum likelihood and 1000 bootstrapped samples [45]. Study goals and the analysis plan were preregistered prior to conducting the research. Results reported here were checked for reproducibility by an independent data analyst (R.M.H.), who recreated the code by working from the manuscript and applying it to a fresh copy of the data set.

## 3. Results

### 3.1. Are Antisocial Behavior Trajectories Associated with Signs of Accelerated Aging by Midlife?

**Figure 1:**
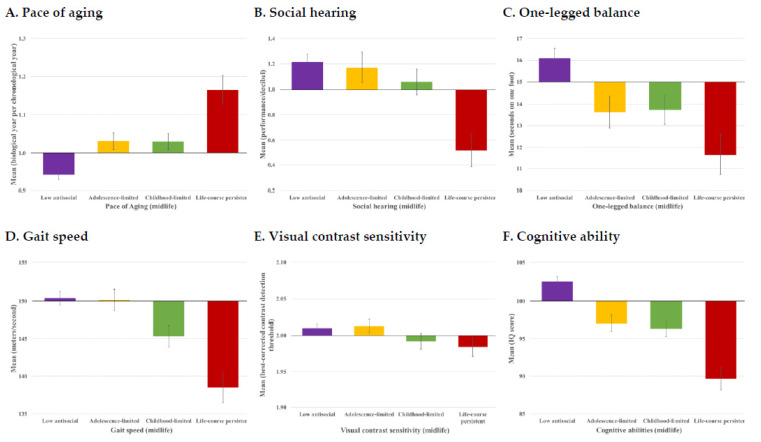
Midlife aging outcomes among individuals following four developmental trajectories of antisocial behavior: Low antisocial (n = 471), Adolescence-limited (n = 173), Childhood-limited (n = 207), and Life-course persistent (n = 83). Each panel shows the mean and standard errors of each measure in original units; group means cross the x-axis set to the overall sample mean.

Study members with a history of antisocial behavior had a significantly faster pace of biological aging by midlife, and this was most evident among those individuals following a LCP antisocial trajectory (Figure 1A, Table 2A; **Model 1**). On average, individuals following the low antisocial behaviors trajectory aged 0.94 (95%CI: 0.92, 0.97) biological year per chronological year. The childhood- and adolescence-limited groups each aged faster, at a rate of 1.03 (95%CI: 0.99, 1.07 for both) biological year per chronological year, on average. By far, the LCP antisocial group aged fastest, on average 1.17 (95%CI: 1.10, 1.24) biological years per chronological year. By age 45, this amounted to an estimated 4.3 extra years of biological aging for Study members following the LCP trajectory compared to low-antisocial trajectory individuals. Individuals following the LCP trajectory also presented at age 45 with poorer social hearing (Table 2B; **Model 1**; β, −0.14, 95%CI, −0.21, −0.08, *p* ≤ 0.001), poorer balance (Table 2C; **Model 1**; β, −0.13, 95%CI, −0.18, −0.06, *p* ≤ 0.001), slower gait speed (Table 2D; **Model 1**; β, −0.18, 95%CI, −0.24, −0.10, *p* ≤ 0.001), and lower cognitive functioning (Table 2F; **Model 1**; β, −0.25, 95%CI, −0.31, −0.18, *p* ≤ 0.001). The only measure on which individuals following a LCP trajectory did not present with significant midlife difficulties was visual contrast sensitivity (Table 2E; **Model 1**; β, −0.06, 95%CI, −0.12, 0.00, *p* = 0.072).

### 3.2. Are the Associations between Antisocial Behavior Trajectories and Signs of Accelerated Aging in Midlife Capturing a Decline in Health?

The association between LCP antisocial behavior and accelerated Pace of Aging was observed even after controlling for poor childhood health, suggesting that the link between antisocial behavior and accelerated aging does not simply reflect developmental origins of poor health already present in childhood (Table 2A; **Model 2**). Similarly, the association between LCP antisocial behavior and the integrity of sensory and motor systems, with the exception of visual contrast sensitivity, were observed even after controlling for their matched childhood health measure, suggesting that the link between LCP antisocial behaviors and the integrity of midlife sensory and motor systems does not simply reflect poor childhood sensory and motor function to begin with (Table 2B–E; **Model 2**). Individuals following the LCP antisocial behavior trajectory also experienced cognitive decline by midlife (Table 2F). On average, these individuals had had the poorest scores on tests of cognitive functioning in childhood (Table 1), and they continued to decline relative to this baseline.

In contrast to the life-course persistent group, individuals following developmentally transient antisocial behavior trajectories (whether CL or AL) showed a less clear picture of compromised integrity of sensory and motor systems at midlife (Table 2). The CL antisocial behavior group presented with accelerated Pace of Aging (Table 2A; **Model 2**) and a decrease in the integrity of motor systems, as measured by one-legged balance (Table 2C; **Model 2**) and gait speed (Table 2D; **Model 2**), after accounting for matched childhood heath measures. The AL group experienced faster Pace of Aging (Table 2A; **Model 2**) as well as a decline in one-legged balance (Table 2C; **Model 2**), after accounting for matched childhood health measures, suggesting that these two associations do not simply reflect poor childhood health to begin with. Individuals in the AL group experienced cognitive decline as well (Table 2F; **Model 2**), and their childhood-to-adulthood cognitive decline was not much different from the decline seen in the life-course persistent group (AL: 3.64 points vs. LCP: 4.19 points). Unlike the LCP group, AL individuals had average scores on tests of cognitive functioning in childhood, while the low-antisocial group performed above-average (Table 1). The CL group also performed poorly on tests of midlife cognitive functioning, but this was a continuation of their poor cognitive functioning in childhood, not a decline relative to their childhood functioning.

### 3.3. Are the Associations between Antisocial Behavior Trajectories and Signs of Accelerated Aging in Midlife Attributable to Health Problems in Adulthood?

**Figure 2:**
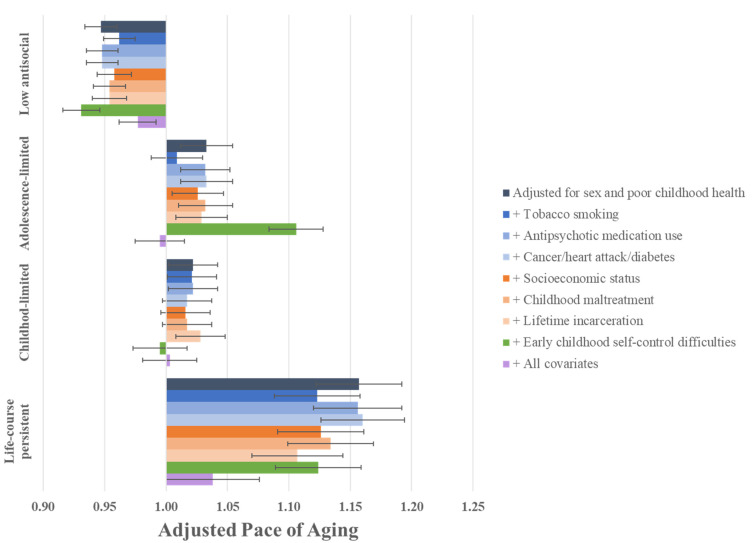
Association between antisocial behavior trajectories and Pace of Aging at midlife, controlling for the 7 study covariates shown in colors (n = 934). The figure compares life-course persistent, childhood-limited, and adolescence-limited antisocial groups to a reference group of study members who engaged in no or low levels of antisocial behavior. Figure shows adjusted means and standard errors for one outcome (Pace of Aging); Supplemental Tables S1–S3 provide the results for all of the other aging outcomes.

Next, we tested whether the antisocial behavior trajectories remained associated with midlife aging outcomes after tobacco smoking, use of antipsychotic medication, and cancer/heart attack/diabetes diagnoses were successively included in our models. Each of these adult health indicators was associated with worse midlife aging outcomes (Table 3). However, none accounted for the association observed between LCP (or AL) antisocial behavior trajectories and accelerated aging (Figure 2 and Supplementary Table S1).

### 3.4. Are the Associations between Antisocial Behavior Trajectories and Signs of Accelerated Aging in Midlife Due to Adverse Experiences Common to Both Antisocial Behavior and Accelerated Aging?

We then tested whether the antisocial behavior trajectories remained associated with midlife aging outcomes after accounting for the socioeconomic origins of the Study members, for their exposure to childhood maltreatment, and for their lifetime incarceration histories. Each of these experiences was associated with worse midlife aging outcomes (Table 3), but none accounted for the association between LCP (or AL) antisocial behavior trajectories and accelerated aging (Figure 2 and Supplementary Table S2).

### 3.5. Are the Associations between Antisocial Behavior Trajectories and Signs of Accelerated Aging in Midlife Due to Shared Risk from Early Childhood Self-Control Difficulties?

Finally, we tested whether the association between antisocial behavior trajectories and midlife aging outcomes could be explained by self-control difficulties that, in particular, characterized toddlers who grew up to be involved in LCP antisocial behavior. Poor early childhood self-control was, indeed, associated with the midlife aging outcomes (Table 1), but it did not statistically account for the association between antisocial behavior trajectories and accelerated aging (Figure 2 and Supplementary Table S3).

### 3.6. Are the Associations between Antisocial Behavior Trajectories and Signs of Accelerated Aging in Midlife Due to the Cumulative Impact of Childhood and Adult Health Problems, and Adverse Experiences?

Our findings reported above showed that, on their own or additively, adult health problems did not account for the association between LCP antisocial behavior trajectory and signs of accelerated aging (Supplementary Table S3, **Model 1**). Similar findings were reported for adverse experiences: individually or additively, they did not account for the link between LCP trajectory and accelerated aging (Supplementary Table S3, **Model 2**). However, the LCP antisocial behavior trajectory was no longer significantly associated with outcomes of accelerated aging when all the covariates were included concomitantly in the model (i.e., corresponding childhood health indicator, tobacco smoking, antipsychotic medication use, cancer/heart attack/diabetes, childhood SES, childhood maltreatment, lifetime incarceration, and early childhood self-control difficulties; Supplementary Table S3, **Model 3**; Figure 2). For example, the standardized association between LCP antisocial behavior and Pace of Aging was 0.22, but when all the covariates were added, this association dropped to 0.06 (*p* ≤ 0.001). Thus, it appears to be the lifelong childhood-to-adulthood lifestyle of LCP individuals that explains their accelerated aging.

### 3.7. Do Individuals on Antisocial Behavior Trajectories Differ in Facial Age?

**Figure 3:**
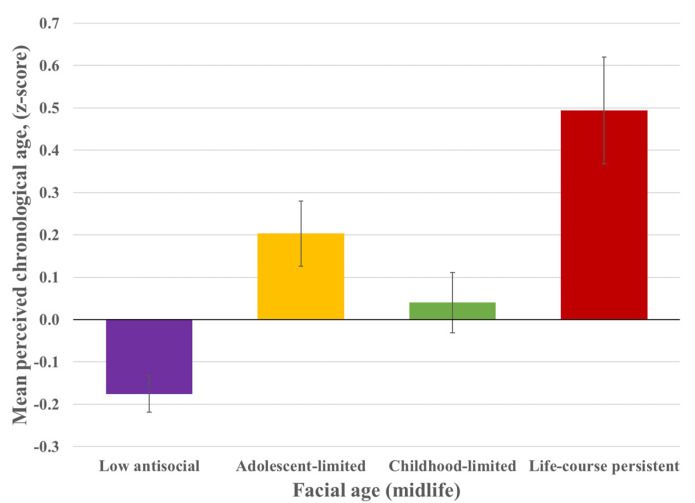
Facial age among individuals following four developmental trajectories of antisocial behavior: Low antisocial (n = 471), Adolescence-limited (n = 173), Childhood-limited (n = 207), and Life-course persistent (n = 83). Panel shows the mean and standard errors of facial age in standardized units.

As a way of summarizing the associations between antisocial behavior trajectories and diverse indicators of accelerated midlife aging, we asked independent raters who had no information about the Study to evaluate facial photographs of the Study members and to estimate their age. The differences were striking: by age 45, individuals following the LCP trajectory of antisocial behavior were rated as looking the oldest (Mean z-score = 0.49, 95%CI: 0.25, 0.75) (Figure 3). To a lesser extent, individuals who followed the CL antisocial behavior trajectory (Mean z-score = 0.04, 95%CI: −0.10, 0.17) and those who followed the AL antisocial behavior trajectory (Mean z-score = 0.20, 95%CI: 0.05, 0.36) were also rated as looking older by midlife. Individuals following the low antisocial behavior trajectory were rated the youngest (Mean z-score = −0.18, 95%CI: −0.26, −0.10). Among LCP and AL offenders, these associations were observed even after accounting for the Study members’ adult health problems (i.e., tobacco smoking, antipsychotic medication use, cancer/heart attack/diabetes) and for their adverse experiences in childhood (e.g., socioeconomic background, maltreatment) and in adulthood (i.e., incarceration) (Supplementary Table S4).

### 3.8. Are There Group Differences in Health Knowledge According to Antisocial Behavior Trajectories?

In addition to having the oldest facial appearance, aging at the fastest pace, and showing the steepest declines in sensory, motor and cognitive systems accompanying biological aging, LCP offenders also had the least health knowledge (mean z-score= −0.73 (95%CI: −0.98, −0.50) vs. low antisocial group mean= 0.22 (95%CI: 0.14, 0.31). Similarly, the CL and the AL had less health knowledge than the low-antisocial group (CL: mean z-score= −0.14 (95%CI: −0.27, 0.00); AL: mean= −0.11 (95%CI: −0.27, 0.03), but both groups were better informed than people following a LCP antisocial behavior trajectory, suggesting that LCP offenders are not well situated to take proactive steps to enhance their health going forward.

### 3.9. Associations between Antisocial Behavior Trajectories and DunedinPACE in Midlife

This longitudinal study relied on multiple indicators of midlife aging, including a measure of the Pace of Aging which observed age-related decline across multiple organ systems over a period of 20 years. Such a measure is not readily available in most other studies, making future replication efforts of the findings reported here difficult. Fortunately, a novel DNA-methylation biomarker of the Pace of Aging has recently been made publicly available, DunedinPACE ([35], Supplementary Methods). We duplicated our tests of the association between antisocial behavior trajectories and the Pace of Aging using DunedinPACE. The results in Supplementary Table S5 (Panel A) show that we obtain results comparable to those of the 19-biomarker longitudinal measure of Pace of Aging (Panel B). This analysis was conducted within the same cohort, so it does not constitute a replication, but rather proof-of-principle that replication tests and extensions of this study are possible in other samples by using this surrogate biomarker.

## 4. Discussion

The aim of this study was to investigate the association between antisocial behavior trajectories and signs of accelerated aging by midlife. This analysis was informed by theoretical perspectives on life-course offending and contemporary work on the link between crime and health [5,24,46,47]. We found evidence to support the hypothesis that individuals engaged in a LCP antisocial behavior trajectory experience an earlier onset and steeper decline in system integrity that underlies biological aging. This link between LCP antisocial behavior and signs of accelerated aging was driven by the cumulative impact of LCP offenders’ background (e.g., lower childhood SES, childhood maltreatment) and lifestyle (e.g., tobacco smoking, incarceration) characteristics, and potentially exacerbated by their limited health literacy. Maintaining health self-reliance for prevention of disability and diseases is an immense challenge for LCP individuals, who are typically characterized by psychological vulnerabilities such as hostility, alienation and antisociality. These individuals typically experience poor social support, exposure to illicit drugs, competing priorities, financial strain [48], stigma, and social exclusion [49]. Moreover, the necessity of maintaining health-promoting behaviors may be somewhat lost on individuals with limited health literacy. This increases the challenges of sustained health-promoting behaviors associated with slower aging, while simultaneously creating wear and tear on the body.

Cumulative disadvantage building from childhood, such as childhood deprivation and disadvantage (e.g., poor childhood health, poverty, childhood maltreatment, early childhood self-control difficulties), may culminate in faster decline in sensory, motor and cognitive functioning accompanying biological aging through processes of biological embedding [23]. Indeed, previous studies report that young adults who show signs of accelerated biological aging are characterized by childhood personal-history risks building from childhood, which were related to patterns of biological wear and tear consistent with faster pace of aging across the life course [25,35,50,51,52]. Simultaneously, habits and behaviors of LCP offenders may further contribute to faster aging through an unhealthy lifestyle, such as tobacco smoking and substance misuse, lack of sleep, physical activity, poor dietary choices, low relationship quality, and exposure to violence and victimization [51,53,54,55,56].

Overall, LCP offenders are more likely than their non-offending peers to impose heavy long-term costs on the healthcare system [46,57]. As medicine is increasingly capable of prolonging lifespan, our results suggest that older adults aging out of crime will likely age into the healthcare system earlier than their chronologically same-aged peers. Consequently, as they age, LCP individuals will represent a disproportionally high cost to the healthcare system. Our findings have clear implications for society: primary prevention targeting health literacy, as well as health promotion interventions that will be needed to support the wellbeing of LCP offenders’ and limit their long-term impact on healthcare and social (e.g., welfare) services. Indeed, these people are hit with double misfortune: they are at highest risk of earlier age-related health decline and they are also the least knowledgeable about positive health behaviors and why they matter.

### Actionable Recommendations: What Can Be Done?

If the association between LCP antisocial lifestyle and aging turns out to be causal, offering health education programs focusing on modifiable health behaviors at every opportunity (e.g., in community-based programs; at school; and at every contact with the judicial, welfare, and healthcare systems) has the potential to slow aging and improve offenders’ health and wellbeing, thereby reducing their burden on healthcare services by limiting risk factors associated with faster age-related health decline because offending starts and peaks long before physical diseases. Diet, for example, has been linked to decelerated biological aging [58,59]. A study of 32,974 warehouse employees indicated that worksites with a wellness program had a higher rate of employees who reported engaging in regular exercise and actively managing their weight [60]. In the US prison context, an investigation of a wellness programs’ effectiveness showed some evidence of reducing depression and smoking for individuals who completed the entire program, although improvements in exercise and nutrition were not evident [61]. These results may be attributable to environmental factors associated with correctional facilities, such as strict rules and regulations as well as poor nutritional offerings. It may be that inmate wellness programs and interventions are beneficial for improving health behaviors through education but institutional policy changes are needed to improve environmental health factors of correctional facilities [61]. Future research is needed to test whether lifestyle interventions can slow the pace of biological aging to improve quality of life in older adults as well as substantial healthcare savings.

LCP individuals start life with disadvantages promoting poor health (e.g., maltreatment) and grow up into a lifestyle that exacerbates their poor health. If the association between LCP antisocial lifestyle and aging is indeed causal, considering antisocial behaviors as a serious health concern could contribute to mitigate the health-span gap between offenders and non-offenders. Offering behavioral interventions on antisocial behavior early in development, before subsequent negative health behaviors are acquired and integrated as part of individuals’ lifestyle to divert potential offenders, especially LCP offenders, onto a new trajectory could reduce the impact of life-course persistent antisocial lifestyle and attendant negative health behaviors and effects [62].

The association between antisocial trajectories and faster aging presents an opportunity for public health to reduce the burden caused by LCP offenders’ early onset of age-related diseases, thereby reducing the upcoming increasingly high costs to the healthcare system. Even if associations between antisocial behaviors and aging are not causal, individuals with a LCP trajectory are a high-priority group to monitor for signs of accelerated aging—such as motor problems and cognitive decline—that become apparent earlier in this group than in the general population. Such monitoring will require greater integration of social and physical health services to reduce health inequalities [14].

Our study has some limitations. First, our analyses were conducted on a single cohort, primarily white New Zealanders. However, it is important to note that studies in the literature show connections between accelerated pace of biological aging, risk factors, and disease outcomes in African American research participants (e.g., [51]). Second, the rate of death among LCP offenders in our sample was relatively low, whereas American studies report elevated rates of early death for LCP offenders [48,63]. Thus, death rates may somewhat differ according to availability of free universal healthcare and degree of firearms control. Third, participants have only been followed to age 45 years, so whether people with signs of accelerated aging by midlife will die younger is not yet known. However, other research has established that our outcome measures, such as Pace of Aging and gait speed, are associated with morbidity and mortality in older adults [12,64]). Moreover, follow-up research will soon be possible within the study sample as a new wave of data collection is scheduled for age 52. Fourth, we did not control for all possible confounders typically associated with LCP offenders’ lifestyle that may be associated with accelerated aging, such as substance misuse. However, substance misuse is inherently part of the antisocial lifestyle, so trying to identify LCP offenders who never misused substances or trying to control statistically for substance misuse history will simply rule out the very lifestyle we are trying to study. Individuals on the life-course-persistent trajectory are characterized by correlated aging and lifestyle features. It is not the case that any one of these features (e.g., Pace of Aging, smoking) “accounts for” or “confounds” the other. Rather, they all contribute to characterize the most persistently antisocial individuals in society. Our study provides a more comprehensive picture of the overall age-related health decline of antisocial individuals and distinguished those on a life-course-persistent trajectory from those on other antisocial trajectories.

## 5. Conclusions

The findings of this cohort study suggest that a trajectory of LCP antisocial behaviors is associated with accelerated aging at midlife, years before the typical onset of age-related diseases. Monitoring of individuals who engage in antisocial behaviors for signs of accelerated aging may have the potential to reduce health inequalities and improve offenders’ lives. Furthermore, study results suggest that juvenile and adult detention center-based health-promoting programs targeting modifiable health-risk behaviors may have the potential to prevent offenders from becoming high-need/high-cost health services users. As the population—including the carceral population—is aging, criminology and geroscience must join forces to foster healthier aging among offenders, thus limiting the growing healthcare costs.

## Figures and Tables

**Table 1:** Health histories, adverse experiences, and self-control difficulties among individuals in the four developmental trajectories of antisocial behaviors. These measures are used as covariates in estimating associations between developmental trajectories of antisocial behavior and aging outcomes.

		Developmental Trajectories of Antisocial Behavior			
		Life-Course Persistent	Childhood- Limited	Adolescence- Limited	Low Antisocial
		(n = 83)	(n = 207)	(n = 173)	(n = 471)
		Means (SD)/%			
**Childhood health indicators**					
	Poor health *	0.15 (1.03)	0.15 (1.02)	−0.03 (1.03)	−0.08 (0.97)
	Hearing *	−0.53 (1.25)	−0.02 (0.94)	0.00 (1.02)	0.10 (0.94)
	Balance *	−0.08 (0.94)	−0.03 (1.00)	0.07 (1.09)	0.00 (0.98)
	Motor development *	−0.12 (1.01)	−0.20 (1.21)	0.07 (0.95)	0.09 (0.90)
	Vision *	0.06 (0.58)	0.01 (0.93)	−0.07 (1.17)	0.01 (1.02)
	Cognitive ability (IQ) *	−0.46 (0.95)	−0.31 (1.04)	0.00 (0.97)	0.22 (0.94)
**Health problems in adulthood**					
	Tobacco smoking	83.1%	52.2%	74.1%	37.8%
	Antipsychotic medication use	2.4%	2.4%	2.3%	1.5%
	Diabetes/heart attack/cancer	4.8%	8.3%	5.8%	5.3%
**Adverse experiences**					
	Childhood socioeconomic status *	−0.64 (0.87)	−0.14 (0.95)	−0.15 (0.97)	0.23 (0.99)
	Childhood maltreatment	27.7%	13.0%	11.0%	3.8%
	Lifetime incarceration	24.1%	2.0%	5.8%	0.6%
Early childhood self-control difficulties *		0.51 (1.30)	0.30 (1.24)	−0.09 (0.86)	−0.19 (0.79)

Note. * Measure standardized to z-score (M = 0, SD = 1). IQ = Intelligence quotient.

**Table 2:** Association between developmental trajectories of antisocial behavior and midlife aging outcomes. Model 1 presents the association adjusted for participants’ sex. Model 2 presents the model adjusted for participants’ sex and each of the corresponding baseline measures of childhood health in order to test whether there has been health decline from early life. Standardized regression coefficients and 95% confidence intervals (CI’s) are presented for all models.

	Model 1	Model 2
	β (95%CI)	
**A. Pace of Aging**		
Life-course persistent	0.22 *** (0.14, 0.28)	0.20 *** (0.13, 0.27)
Childhood-limited	0.12 *** (0.06, 0.20)	0.11 ** (0.04, 0.17)
Adolescence-limited	0.12 *** (0.05, 0.18)	0.11 ** (0.05, 0.18)
Poor childhood health		0.20 *** (0.13, 0.27)
**B. Social hearing**		
Life-course persistent	−0.14 *** (−0.21, −0.08)	−0.13 *** (−0.19, −0.07)
Childhood-limited	−0.05 (−0.12, 0.02)	−0.04 (−0.11, 0.03)
Adolescence-limited	−0.01 (−0.09, 0.06)	−0.01 (−0.09, 0.06)
Childhood hearing		0.09 (0.00, 0.18)
**C. One-legged balance**		
Life-course persistent	−0.13 *** (−0.18, −0.06)	−0.13 *** (−0.18, −0.06)
Childhood-limited	−0.10 ** (−0.17, −0.03)	−0.10 ** (−0.17, −0.03)
Adolescence-limited	−0.10 ** (−0.16, −0.03)	−0.10 ** (−0.17, −0.04)
Childhood balance		0.15 *** (0.08, 0.21)
**D. Gait speed**		
Life-course persistent	−0.18 *** (−0.24, −0.10)	−0.16 *** (−0.21, −0.09)
Childhood-limited	−0.11 ** (−0.18, −0.04)	−0.08 * (−0.15, −0.01)
Adolescence-limited	−0.01 (−0.07, 0.06)	0.00 (−0.07, 0.06)
Childhood motor development		0.28 *** (0.21, 0.35)
**E. Visual contrast sensitivity**		
Life-course persistent	−0.06 (−0.12, 0.00)	−0.06 (−0.12, 0.00)
Childhood-limited	−0.06 (−0.13, 0.02)	−0.06 (−0.13, 0.02)
Adolescence-limited	0.01 (−0.05, 0.07)	0.01 (−0.05, 0.08)
Childhood vision		0.12 * (0.02, 0.23)
**F. Cognitive functioning**		
Life-course persistent	−0.25 *** (−0.31, −0.18)	−0.09 *** (−0.13, −0.04)
Childhood-limited	−0.17 *** (−0.24, −0.11)	−0.01 (−0.06, 0.03)
Adolescence-limited	−0.14 *** (−0.21, −0.07)	−0.07 ** (−0.12, −0.03)
Childhood IQ		0.77 *** (0.74, 0.80)

**Note.** Analyses compare each antisocial group to the reference group “low antisocial behavior.” **Pace of aging** was measured with repeated assessments of a panel of 19 biomarkers taken at ages 26, 32, 38, and 45 years. **Social hearing** was assessed using the Listening in Spatialized Noise Sentences Test (LiSN-S; Pohank, Switzerland). **One-legged balance** was measured using the Unipedal Stance Test, as the maximum time achieved across 3 trials of the test with eyes closed. **Gait speed** was assessed with the 6-m-long GAITRite Electronic Walkway (CIR Systems, Inc). **Visual contrast sensitivity** was assessed using the Thomson Test Chart (2016). **Cognitive functioning (IQ)** was measured using the Wechsler Adult Intelligence Scale-IV (Wechsler, 2008). *** *p* ≤ 0.001; ** *p* ≤ 0.01; * *p* ≤ 0.05.

**Table 3:** Correlations between midlife aging outcomes and health problems in adulthood, adverse experiences and self-control difficulties (n = 934).

	Health Problems in Adulthood			Adverse Experiences			Early Childhood Self-Control Difficulties
	Tobacco Smoking	Antipsychotic Medication Use	Cancer/ Heart Attack/Diabetes	Childhood SES	Childhood Maltreatment	Lifetime Incarceration	
Pace of Aging	0.23 ***	0.19 ***	0.18 ***	−0.24 ***	0.19 ***	0.23 ***	0.15 ***
Social Hearing	−0.11 ***	−0.10 **	−0.04	0.13 ***	−0.08 *	−0.11 ***	−0.18 ***
One-legged Balance	−0.16 ***	−0.12 ***	−0.12 ***	0.19 ***	−0.14 ***	−0.08 **	−0.15 ***
Gait Speed	−0.08 *	−0.09 *	−0.12 **	0.20 ***	−0.14 ***	−0.11 ***	−0.17 ***
Visual Contrast Sensitivity	−0.07 *	−0.12 **	−0.03	0.06	−0.14 ***	0.02	−0.10 **
Cognitive Functioning	−0.2 1***	−0.15 ***	−0.07 *	0.36 ***	−0.17 ***	−0.15 ***	−0.33 ***

**Note. Pace of aging** was measured with repeated assessments of a panel of 19 biomarkers taken at ages 26, 32, 38, and 45 years. **Social hearing** was assessed using the Listening in Spatialized Noise Sentences Test (LiSN-S; Pohank, Switzerland). **One-legged balance** was measured using the Unipedal Stance Test, as the maximum time achieved across 3 trials of the test with eyes closed. **Gait speed** was assessed with the 6-m-long GAITRite Electronic Walkway (CIR Systems, Inc). **Visual contrast sensitivity** was assessed using the Thomson Test Chart (2016). **Cognitive functioning (IQ)** was measured using the Wechsler Adult Intelligence Scale-IV (Wechsler, 2008). *** *p* ≤ 0.001; ** *p* ≤ 0.01; * *p* ≤ 0.05.

## Data Availability

The Dunedin Study data are available on request to TEM by qualified scientists. Requests require a concept paper describing the purpose of data access, ethical approval at the applicant’s institution, and provision for secure data access. We offer secure access on the Duke University, Otago University, and King’s College London campuses. All data analysis scripts and results files are available for review.

## Supplementary Materials

The following supporting information can be downloaded at: https://www.mdpi.com/article/10.3390/ijerph192114402/s1. Supplementary Methods, Attrition Analyses. Supplementary Results: Table S1: Association between antisocial behavior trajectories and aging outcomes at age 45 adjusted for participants’ health problems in adulthood (n = 934); Table S2. Association between antisocial behavior trajectories and aging outcomes at age 45 adjusted for adverse experiences and early childhood self-control difficulties (n = 934); Table S3. Association between antisocial behavior trajectories and aging outcomes at age 45 adjusted for corresponding childhood health measures, adult health problems, adverse experiences and early childhood self-control difficulties (n = 934); Table S4. Association between antisocial behavior trajectories and facial age (n = 934); Table S5. Association between antisocial behavior trajectories and accelerated Pace of Aging as measured by DunedinPACE and PoA (n = 934). References [39,65,66,67,68,69,70,71,72,73,74,75,76,77,78,79,80,81,82,83,84,85] are cited in the supplementary material.
