# Supplementary material for: Life-Course Persistent Antisocial Behavior and Accelerated Biological Aging in a Longitudinal Birth Cohort
Source: Int J Environ Res Public Health. 2022 Nov 3;19(21):14402. doi: 10.3390/ijerph192114402 (PMC9657643; doi:10.3390/ijerph192114402)
Supplement: Supplementary file 1 [file ijerph-19-14402-s001.zip › ijerph-1975281-supplementary.pdf]

Life-course persistent antisocial behavior and accelerated biological aging in a longitudinal birth cohort

Langevin, S. et al.

Supplementary Results………………………………………………………………… **p.2-12**

Supplementary Table S1. Association between antisocial behavior trajectories and aging outcomes at age 45 adjusted for participants' health problems in adulthood (n=934).

Supplementary Table S2. Association between antisocial behavior trajectories and aging outcomes at age 45 adjusted for adverse experiences and early childhood self-control difficulties (n=934).

Supplementary Table S3. Association between antisocial behavior trajectories and aging outcomes at age 45 adjusted for corresponding childhood health measures, adult health problems, adverse experiences and early childhood self-control difficulties (n=934).

Supplementary Table S4. Association between antisocial behavior trajectories and facial age (n=934).

Supplementary Table S5. Association between antisocial behavior trajectories and accelerated Pace of Aging as measured by DunedinPACE and PoA (n=934).

Attrition Analyses……………………………………………………………….. p. 13-14

Supplementary Methods…………………………………………………………... p. 15-22

Supplementary Table S1. Association between antisocial behavior trajectories and aging outcomes at age 45 adjusted for participants' health problems in adulthood (n=934). All analyses were adjusted for participants' sex. Model 1 presents associations adjusted for participants' sex and each of the corresponding baseline measures of childhood health, and serves as a comparison model. Model 2 presents associations adjusted for each of the corresponding baseline measures of childhood health and tobacco smoking. Model 3 presents associations adjusted for each of the corresponding baseline measures of childhood health and antipsychotic medication use. Model 4 presents associations adjusted for each of the corresponding baseline measures of childhood health and cancer/heart attack/diabetes diagnosis up to age 45.

	Model 1	Model 2	Model 3	Model 4
	β (95% Cl)			
**A. Pace of Aging**				
Life-course persistent	.20*** (.13, .27)	.16*** (.08, .22)	.20*** (.13, .27)	.20*** (.13, .27)
Childhood-limited	.11** (.04, .17)	.08* (.02, .15)	.10** (.04, .17)	.10** (.03, .16)
Adolescence-limited	.11*** (.05, .18)	.06 (.00, .13)	.11*** (.05, .17)	.11*** (.05, .18)
Poor childhood health	.20*** (.13, .27)	.20*** (.14, .27)	.19*** (.13, .26)	.19*** (.12, .26)
Tobacco smoking		.18*** (.12, .24)		
Antipsychotic medication use			.17** (.06, .28)	
Cancer / heart attack / diabetes				.17*** (.09, .24)
**B. Social hearing**				
Life-course persistent	-.13*** (-.19, -.07)	-.10** (-.17, -.03)	-.13*** (-.19, -.06)	-.13*** (-.19, -.07)
Childhood-limited	-.04 (-.11, .03)	-.03 (-.10, .04)	-.04 (-.11, .03)	-.04 (-.11, .03)
Adolescence-limited	-.01 (-.09, .06)	.02 (-.06, .10)	-.01 (-.08, .06)	-.01 (-.09, .06)
Childhood hearing	.09 (.00, .18)	.09 (.00, .18)	.09 (.00, .18)	.09 (.00, .18)
Tobacco smoking		-.09** (-.17, -.02)		
Antipsychotic medication use			-.09* (-.16, -.01)	
Cancer / heart attack / diabetes				-.04 (-.09, .03)
**C. One-legged balance**				
Life-course persistent	-.13*** (-.18, -.06)	-.09** (-.15, -.03)	-.13*** (-.18, -.06)	-.13*** (-.18, -.06)
Childhood-limited	-.10** (-.17, -.03)	-.09* (-.15, -.02)	-.10** (-.17, -.04)	-.10** (-.16, -.03)
Adolescence-limited	-.10** (-.17, -.04)	-.07 (-.13, .01)	-.10** (-.17, -.03)	-.10** (-.17, -.03)
Childhood balance	.15*** (.08, .21)	.15*** (.08, .21)	.15*** (.08, .21)	.15*** (.08, .21)
Tobacco smoking		-.13*** (-.20, -.06)		
Antipsychotic medication use			-.12*** (-.16, -.07)	
Cancer / heart attack / diabetes				-.11*** (-.17, -.05)
**D. Gait speed**				
Life-course persistent	-.16*** (-.21, -.09)	-.15*** (-.20, -.07)	-.15*** (-.21, -.09)	-.16*** (-.21, -.09)
Childhood-limited	-.08* (-.15, -.01)	-.08* (-.15, .00)	-.08* (-.15, -.01)	-.08* (-.15, -.01)
Adolescence-limited	.00 (-.07, .06)	.01 (-.07, .07)	.00 (-.07, .06)	.00 (-.07, .06)
Childhood motor development	.28*** (.21, .35)	.28*** (.21, .35)	.28*** (.21, .35)	.27*** (.20, .34)
Tobacco smoking		-.04 (-.11, .03)		
Antipsychotic medication use			-.07 (-.15, .01)	
Cancer / heart attack / diabetes				-.09** (-.16, -.03)

	Model 1	Model 2	Model 3	Model 4
		β (95% Cl)		
**E. Visual contrast sensitivity**				
Life-course persistent	-.06 (-.12, .00)	-.04 (-.10, .02)	-.05 (-.11, .01)	-.06 (-.11, .00)
Childhood-limited	-.06 (-.13, .02)	-.05 (-.12, .03)	-.05 (-.13, .02)	-.05 (-.13, .02)
Adolescence-limited	.01 (-.05, .08)	.03 (-.03, .10)	.02 (-.04, .08)	.01 (-.04, .08)
Childhood vision	.12* (.02, .23)	.12* (.02, .22)	.12* (.02, .22)	.12* (.02, .23)
Tobacco smoking		-.08* (-.14, -.01)		
Antipsychotic medication use			-.12** (-.20, -.03)	
Cancer / heart attack / diabetes				-.03 (-.10, .05)
**F. Cognitive functioning**				
Life-course persistent	-.09*** (-.13, -.04)	-.07** (-.11, -.02)	-.09*** (-.13, -.04)	-.09*** (-.13, -.04)
Childhood-limited	-.01 (-.06, .03)	.00 (-.05, .04)	-.01 (-.06, .03)	-.01 (-.06, .03)
Adolescence-limited	-.07*** (-.12, -.03)	-.05* (-.10, -.01)	-.07*** (-.12, -.03)	-.07*** (-.12, -.03)
Childhood IQ	.77*** (.74, .80)	.76*** (.73, .79)	.76*** (.73, .79)	.77*** (.73, .80)
Tobacco smoking		-.07*** (-.12, -.03)		
Antipsychotic medication use			-.09** (-.15, -.03)	
Cancer / heart attack / diabetes				-.03 (-.07, .01)

Notes. Standardized regression coefficients and 95% confidence intervals are presented for all models. Analyses compare each antisocial group to the reference group “low antisocial behaviors”. **Pace of Aging** was measured with repeated assessments of a panel of 19 biomarkers taken at ages 26, 32, 38, and 45 years. **Social hearing** was assessed using the Listening in Spatialised Noise–Sentences Test (LiSN-S; Phonak, Switzerland). **One-legged balance** was measured using the Unipedal Stance Test, as the maximum time achieved across 3 trials of the test with eyes closed. **Gait speed** was assessed with the 6-m-long GAITRite Electronic Walkway (CIR Systems, Inc). **Visual contrast sensitivity** was assessed using the Thomson Test Chart (2016). **Cognitive functioning (IQ)** was measured using the Wechsler Adult Intelligence Scale-IV (Wechsler, 2008). ***p ≤ .001, ** p ≤ .01, * p ≤ .05

Supplementary Table S2. Association between antisocial behavior trajectories and aging outcomes at age 45 adjusted for adverse experiences and early childhood self-control difficulties (n=934). All analyses were adjusted for participants' sex. Model 1 presents associations adjusted for each of the corresponding baseline measures of childhood health, and serves as a comparison model. Model 2 presents associations adjusted for each of the corresponding baseline measures of childhood health and childhood socioeconomic status. Model 3 presents associations adjusted for each of the corresponding baseline measures of childhood health and childhood maltreatment. Model 4 presents associations adjusted for each of the corresponding baseline measures of childhood health and lifetime incarceration. Model 5 presents associations adjusted for each of the corresponding baseline measures of childhood health and early childhood self-control difficulties.

	Model 1	Model 2	Model 3<br>β (95% Cl)	Model 4	Model 5
**A. Pace of aging**					
Life-course persistent	.20*** (.13, .27)	.16*** (.09, .23)	.17*** (.10, .25)	.15*** (.07, .22)	.19*** (.12, .26)
Childhood-limited	.11** (.04, .17)	.08* (.02, .15)	.09** (.03, .15)	.10** (.04, .17)	.09** (.02, .16)
Adolescence-limited	.11*** (.05, .18)	.09** (.03, .16)	.10** (.04, .17)	.10** (.04, .16)	.11*** (.05, .18)
Poor childhood health	.20*** (.13, .27)	.19*** (.12, .25)	.20*** (.13, .27)	.19*** (.12, .26)	.19*** (.12, .26)
Childhood socioeconomic status		-.17*** (-.23, -.11)			
Childhood maltreatment			.10** (.03, .17)		
Lifetime incarceration				.17*** (.07, .26)	
Early self-control difficulties					.09* (.02, .17)
**B. Social hearing**					
Life-course persistent	-.13*** (-.19, -.07)	-.10** (-.17, -.04)	-.12*** (-.19, -.05)	-.10** (-.17, -.04)	-.10** (-.17, -.04)
Childhood-limited	-.04 (-.11, .03)	-.03 (-.10, .05)	-.04 (-.11, .04)	-.04 (-.11, .03)	-.02 (-.09, .05)
Adolescence-limited	-.01 (-.09, .06)	.01 (-.07, .08)	-.01 (-.08, .07)	.00 (-.08, .07)	.00 (-.08, .07)
Childhood hearing	.09 (.00, .18)	.08 (-.01, .17)	.09 (-.01, .18)	.09 (.00, .18)	.08 (-.01, .17)
Childhood socioeconomic status		.10** (.03, .18)			
Childhood maltreatment			-.03 (-.09, .04)		
Lifetime incarceration				-.08** (-.14, -.02)	
Early self-control difficulties					-.15*** (-.21, -.08)
**C. One-legged balance**					
Life-course persistent	-.13*** (-.18, -.06)	-.09** (-.15, -.02)	-.11*** (-.17, -.04)	-.11*** (-.16, -.04)	-.11*** (-.16, -.04)
Childhood-limited	-.10** (-.17, -.03)	-.08* (-.15, -.01)	-.09** (-.16, -.02)	-.10** (-.17, -.03)	-.08* (-.15, -.01)
Adolescence-limited	-.10** (-.17, -.04)	-.08* (-.14, -.01)	-.10** (-.16, -.03)	-.10** (-.16, -.03)	-.10** (-.16, -.03)

	Model 1	Model 2	Model 3<br>β (95% CI)	Model 4	Model 5
Childhood balance	.15*** (.08, .21)	.13*** (.07, .20)	.14*** (.07, .21)	.15*** (.08, .21)	.13*** (.06, .20)
Childhood socioeconomic status		.15*** (.09, .22)			
Childhood maltreatment			-.06* (-.12, .00)		
Lifetime incarceration				-.06 (-.13, .00)	
Early self-control difficulties					-.10*** (-.16, -.04)
**D. Gait speed**					
Life-course persistent	-.16*** (-.21, -.09)	-.12*** (-.19, -.06)	-.14*** (-.20, -.07)	-.13*** (-.20, -.07)	-.15*** (-.21, -.08)
Childhood-limited	-.08* (-.15, -.01)	-.06 (-.13, .01)	-.07* (-.14, .00)	-.08* (-.15, -.01)	-.07* (-.14, .00)
Adolescence-limited	.00 (-.07, .06)	.02 (-.06, .08)	.00 (-.07, .07)	.00 (-.07, .07)	.00 (-.07, .06)
Childhood motor development	.28*** (.21, .35)	.26*** (.18, .33)	.28*** (.21, .35)	.28*** (.21, .35)	.26*** (.18, .34)
Childhood socioeconomic status		.13*** (.07, .20)			
Childhood maltreatment			-.05 (-.11, .02)		
Lifetime incarceration				-.07* (-.13, -.01)	
Early self-control difficulties					-.05 (-.12, .02)
**E. Visual contrast sensitivity**					
Life-course persistent	-.06 (-.12, .00)	-.05 (-.10, .01)	-.03 (-.09, .04)	-.07* (-.14, -.01)	-.04 (-.10, .02)
Childhood-limited	-.06 (-.13, .02)	-.05 (-.12, .02)	-.04 (-.11, .04)	-.06 (-.13, .02)	-.04 (-.12, .04)
Adolescence-limited	.01 (-.05, .08)	.02 (-.04, .08)	.03 (-.03, .09)	.01 (-.05, .07)	.02 (-.04, .08)
Childhood vision	.12* (.02, .23)	.12* (.02, .23)	.11* (.02, .23)	.11* (.02, .22)	.11 (.01, .22)
Childhood socioeconomic status		.05 (-.02, .11)			
Childhood maltreatment			-.11** (-.18, -.04)		
Lifetime incarceration				.05 (-.01, .11)	
Early self-control difficulties					-.08* (-.15, -.01)
**F. Cognitive functioning**					
Life-course persistent	-.09*** (-.13, -.04)	-.08*** (-.12, -.04)	-.08*** (-.12, -.04)	-.07** (-.11, -.02)	-.08*** (-.12, -.04)
Childhood-limited	-.01 (-.06, .03)	-.01 (-.06, .03)	-.01 (-.05, .04)	-.01 (-.06, .03)	-.01 (-.05, .04)
Adolescence-limited	-.07*** (-.12, -.03)	-.07** (-.12, -.03)	-.07** (-.12, -.03)	-.07** (-.11, -.03)	-.07*** (-.12, -.03)
Childhood IQ	.77*** (.74, .80)	.75*** (.72, .79)	.77*** (.73, .80)	.77*** (.73, .80)	.75*** (.72, .79)
Childhood socioeconomic status		.03 (-.01, .08)			
Childhood maltreatment			-.02 (-.06, .02)		

	Model 1	Model 2	Model 3<br>β (95% Cl)	Model 4	Model 5
Lifetime incarceration				-.05 (-.11, .00)	
Early self-control difficulties					-.04 (-.09, .00)

Notes. Standardized regression coefficients and 95% confidence intervals are presented for all models. Analyses compare each antisocial group to the reference group "low antisocial behaviors". **Pace of Aging** was measured with repeated assessments of a panel of 19 biomarkers taken at ages 26, 32, 38, and 45 years. **Social hearing** was assessed using the Listening in Spatialised Noise–Sentences Test (LiSN-S; Phonak, Switzerland). **One-legged balance** was measured using the Unipedal Stance Test, as the maximum time achieved across 3 trials of the test with eyes closed. **Gait speed** was assessed with the 6-m-long GAITRite Electronic Walkway (CIR Systems, Inc). **Visual contrast sensitivity** was assessed using the Thomson Test Chart (2016). **Cognitive functioning (IQ)** was measured using the Wechsler Adult Intelligence Scale-IV (Wechsler, 2008).
***p≤.001, ** p≤..01, * p≤.05

Supplementary Table S3. Association between antisocial behavior trajectories and aging outcomes at age 45 adjusted for corresponding childhood health, adult health problems, adverse experiences and early childhood self-control difficulties (n=934). All analyses were adjusted for participants' sex. Model 1 presents associations adjusted for each of the corresponding baseline measures of childhood health and adult health problems. Model 2 presents associations adjusted for each of the corresponding baseline measures of childhood health and adverse experiences. Model 3 presents associations adjusted for each of the corresponding baseline measures of childhood health, adult health problems, adverse experiences, and early childhood self-control difficulties.

	Model 1	Model 2	Model 3
		β (95% Cl)	
**A. Pace of aging**			
Life-course persistent	.15*** (.08, .22)	.09* (.01, .17)	.06 (-.02, .14)
Childhood-limited	.07* (.01, .14)	.07* (.00, .13)	.04 (-.02, .10)
Adolescence-limited	.06 (-.01, .12)	.07* (.00, .13)	.03 (-.04, .09)
Poor childhood health	.18*** (.12, .25)	.18*** (.11, .25)	.16*** (.09, .22)
Tobacco smoking	.18*** (.12, .24)		.15*** (.09, .21)
Antipsychotic medication use	.17** (.06, .28)		.17** (.06, .27)
Cancer / heart attack / diabetes	.17*** (.09, .25)		.16*** (.09, .23)
Childhood socioeconomic status		-.15*** (-.21, -.10)	-.14*** (-.20, -.09)
Childhood maltreatment		.08* (.02, .15)	.07* (.01, .14)
Lifetime incarceration		.16*** (.06, .26)	.13** (.04, .22)
Early self-control difficulties			.03 (-.03, .10)
**B. Social hearing**			
Life-course persistent	-.10** (-.17, -.03)	-.08* (-.15, -.01)	-.05 (-.12, .03)
Childhood-limited	-.03 (-.10, .04)	-.02 (-.10, .05)	.01 (-.06, .08)
Adolescence-limited	.02 (-.06, .10)	.02 (-.06, .09)	.04 (-.05, .11)
Childhood hearing	.09 (.00, .18)	.09 (-.01, .17)	.08 (-.01, .17)
Tobacco smoking	-.09** (-.17, -.02)		-.07* (-.15, .00)
Antipsychotic medication use	-.09* (-.16, -.01)		-.08 (-.15, .01)
Cancer / heart attack / diabetes	-.04 (-.09, .02)		-.02 (-.08, .03)
Childhood socioeconomic status		.09* (.02, .17)	.09* (.02, .17)
Childhood maltreatment		-.02 (-.08, .05)	.00 (-.06, .07)
Lifetime incarceration		-.07* (-.13, -.01)	-.05 (-.11, .01)
Early self-control difficulties			-.13*** (-.19, -.06)
**C. One-legged balance**			
Life-course persistent	-.09** (-.15, -.03)	-.06 (-.12, .01)	-.03 (-.10, .04)
Childhood-limited	-.08* (-.15, -.01)	-.07* (-.14, .00)	-.04 (-.11, .03)
Adolescence-limited	-.06 (-.13, .01)	-.07* (-.13, .00)	-.04 (-.10, .04)
Childhood balance	.15*** (.08, .21)	.13*** (.07, .20)	.12*** (.06, .18)
Tobacco smoking	-.13*** (-.20, -.06)		-.11** (-.18, -.05)
Antipsychotic medication use	-.11*** (-.15, -.07)		-.11*** (-.16, -.06)
Cancer / heart attack / diabetes	-.11*** (-.17, -.05)		-.10*** (-.16, -.05)
Childhood socioeconomic status		.14*** (.08, .21)	.13*** (.07, .20)
Childhood maltreatment		-.05 (-.12, .01)	-.04 (-.10, .02)
Lifetime incarceration		-.05 (-.12, .01)	-.02 (-.08, .04)

	Model 1	Model 2	Model 3
		β (95% Cl)	
Early self-control difficulties			-.08* (-.14, -.01)
**D. Gait speed**			
Life-course persistent	-.14*** (-.21, -.07)	-.10** (-.16, -.03)	-.09* (-.15, -.02)
Childhood-limited	-.07* (-.15, .00)	-.06 (-.13, .01)	-.05 (-.12, .02)
Adolescence-limited	.01 (-.06, .07)	.03 (-.04, .09)	.03 (-.04, .10)
Childhood motor development	.27*** (.19, .34)	.26*** (.18, .33)	.23*** (.15, .31)
Tobacco smoking	-.04 (-.11, .02)		-.02 (-.09, .04)
Antipsychotic medication use	-.07 (-.15, .01)		-.07 (-.15, .01)
Cancer / heart attack / diabetes	-.09** (-.16, -.03)		-.09* (-.16, -.02)
Childhood socioeconomic status		.13*** (.06, .19)	.13*** (.06, .19)
Childhood maltreatment		-.03 (-.10, .02)	-.03 (-.10, .03)
Lifetime incarceration		-.06* (-.12, .00)	-.05 (-.11, .01)
Early self-control difficulties			-.04 (-.11, .03)
**E. Visual contrast sensitivity**			
Life-course persistent	-.04 (-.10, .02)	-.03 (-.10, .03)	-.01 (-.08, .05)
Childhood-limited	-.04 (-.11, .03)	-.03 (-.10, .04)	-.01 (-.09, .06)
Adolescence-limited	.04 (-.03, .10)	.03 (-.04, .09)	.05 (-.02, .11)
Childhood vision	.12* (.02, .22)	.11* (.02, .23)	.11* (.01, .22)
Tobacco smoking	-.07* (-.14, -.01)		-.07* (-.14, .00)
Antipsychotic medication use	-.12** (-.20, -.03)		-.12** (-.20, -.03)
Cancer / heart attack / diabetes	-.03 (-.10, .05)		-.03 (-.10, .05)
Childhood socioeconomic status		.04 (-.02, .11)	.04 (-.03, .10)
Childhood maltreatment		-.10** (-.18, -.03)	-.09** (-.17, -.03)
Lifetime incarceration		.05 (-.01, .11)	.07* (.01, .13)
Early self-control difficulties			-.06 (-.13, .01)
**F. Cognitive functioning**			
Life-course persistent	-.07** (-.11, -.02)	-.06* (-.10, -.01)	-.05 (-.09, .00)
Childhood-limited	.00 (-.05, .04)	-.01 (-.05, .04)	.01 (-.04, .05)
Adolescence-limited	-.05* (-.10, -.01)	-.06** (-.11, -.02)	-.05* (-.09, .00)
Childhood IQ	.75*** (.72, .78)	.75*** (.71, .79)	.73*** (.69, .77)
Tobacco smoking	-.07*** (-.12, -.03)		-.06** (-.11, -.02)
Antipsychotic medication use	-.09** (-.15, -.03)		-.09** (-.15, -.03)
Cancer / heart attack / diabetes	-.03 (-.07, .01)		-.03 (-.06, .01)
Childhood socioeconomic status		.03 (-.02, .08)	.04 (-.01, .08)
Childhood maltreatment		-.02 (-.06, .02)	-.01 (-.05, .03)
Lifetime incarceration		-.05 (-.10, .00)	-.04 (-.08, .01)
Early self-control difficulties			-.03 (-.08, .01)

Notes. Standardized regression coefficients and 95% confidence intervals are presented for all models. Analyses compare each antisocial group to the reference group "low antisocial behaviors". **Pace of Aging** was measured with repeated assessments of a panel of 19 biomarkers taken at ages 26, 32, 38, and 45 years. **Social hearing** was assessed using the Listening in Spatialised Noise–Sentences Test (LiSN-S; Phonak, Switzerland). **One-legged balance** was

measured using the Unipedal Stance Test, as the maximum time achieved across 3 trials of the test with eyes closed. **Gait speed** was assessed with the 6-m-long GAITRite Electronic Walkway (CIR Systems, Inc). **Visual contrast sensitivity** was assessed using the Thomson Test Chart (2016). **Cognitive functioning (IQ)** was measured using the Wechsler Adult Intelligence Scale-IV (Wechsler, 2008). ***p≤.001, ** p≤..01, * p≤.05

Supplementary Table S4. Association between antisocial behavior trajectories and facial age. Model 1 presents associations adjusted for participants' sex, and serves as a comparison model. Model 2 presents associations adjusted for participants' sex and tobacco smoking. Model 3 presents associations adjusted for participants' sex and antipsychotic medication use. Model 4 presents associations adjusted for participants' sex and cancer/heart attack/diabetes diagnosis up to age 45. Model 5 presents associations adjusted for participants' sex and childhood socioeconomic status. Model 6 presents associations adjusted for participants' sex and childhood maltreatment. Model 7 presents associations adjusted for participants' sex and lifetime incarceration. Model 8 presents associations adjusted for participants' sex and early childhood self-control difficulties.

	Model 1	Model 2	Model 3	Model 4	Model 5	Model 6	Model 7	Model 8
	β (95% Cl)							
**Facial age**								
Life-course persistent	.19*** (.12, .27)	.15***(.08, .23)	.19***(.11, .26)	.19***(.12, .26)	.14***(.07, .22)	.17***(.10, .25)	.15***(.07, .23)	.17***(.09, .24)
Childhood-limited	.09** (.02, .16)	.07* (.00, .14)	.09** (.02, .15)	.09* (.02, .15)	.06 (-.01, .12)	.08* (.01, .14)	.09* (.02, .15)	.07 (.00, .13)
Adolescence-limited	.15*** (.08, .22)	.10** (.04, .17)	.15***(.08, .22)	.15***(.08, .21)	.12***(.05, .19)	.14***(.08, .21)	.14***(.07, .20)	.14***(.08, .21)
Tobacco smoking		.16***(.09, .22)						
Antipsychotic medication use			.08* (.02.15)					
Cancer/heart attack/diabetes				.09***(.04, .14)				
Childhood socioeconomic status					-.19***(-.25,-.12)			
Childhood maltreatment						.06 (-.01, .12)		
Lifetime incarceration							.13***(.06, .19)	
Early self-control difficulties								.11** (.04, .19)

Notes. Standardized regression coefficients and 95% confidence intervals are presented for all models. Analyses compare each antisocial group to the reference group "low antisocial behaviors". **Facial age** was measured by a panel of 8 independent raters using standardized (non-smiling) facial photographs using a Likert scale to categorize participants into a 5-year age range and a 7-item Likert scale to assign a "relative age" to each participant (1= "looking young", 7= "looking old"). Facial age was derived by standardizing and averaging these two scores. ***p ≤ .001, ** p ≤ .01, * p ≤ .05

Supplementary Table S5. Association between antisocial behavior trajectories and accelerated Pace of Aging. Panel A presents results using DunedinPACE, a novel single-time-point measure that quantifies Pace of Aging from a blood test. Panel B presents the original results presented in the Main Article, using Pace of Aging (a composite measure of 19 biomarkers measured repeatedly from ages 26-45 years) as the outcome variable.

	Model 1	Model 2	Model 3	Model 4	Model 5	Model 6	Model 7	Model 8
	β (95% Cl)							
**A. Dunedin PACE**								
Life-course persistent	.23***(.16, .30)	.18***(.11, .24)	.23***(.16, .30)	.23***(.16, .30)	.19***(.12, .26)	.19***(.12, .26)	.18***(.11, .25)	.21***(.14, .28)
Childhood-limited	.11***(.05, .18)	.09* (.02, .15)	.12***(.05, .18)	.11** (.04, .17)	.09** (.02, .15)	.09** (.03, .16)	.11***(.04, .17)	.09** (.02, .16)
Adolescence-limited	.13***(.06, .20)	.07* (.00, .14)	.13***(.06, .20)	.13***(.06, .20)	.11**(.04, .18)	.12***(.05, .19)	.12***(.05, .19)	.13***(.06, .20)
Poor childhood health	.19***(.12, .26)	.19***(.12, .26)	.19***(.12, .25)	.18***(.11, .25)	.18***(.11, .24)	.19***(.12, .26)	.18***(.11, .25)	.18***(.11, .24)
Tobacco smoking		.22***(.15, .28)						
Antipsychotic medication use			.15**(.05, .24)					
Cancer / heart attack / diabetes				.15***(.08, .22)				
Childhood socioeconomic status					-.18***(-.24, -.12)			
Childhood maltreatment						.14***(.07, .21)		
Lifetime incarceration							.18***(.08, .27)	
Early self-control difficulties								.13***(.06, .19)
**B. Pace of aging**								
Life-course persistent	.20***(.13, .27)	.16***(.08, .22)	.20***(.13, .27)	.20***(.13, .27)	.16***(.09, .23)	.17***(.10, .25)	.15***(.07, .22)	.19***(.12, .26)
Childhood-limited	.11** (.04, .17)	.08** (.02, .15)	.10** (.04, .17)	.10** (.03, .16)	.08* (.02, .15)	.09** (.03, .15)	.10** (.04, .17)	.09** (.02, .16)
Adolescence-limited	.11***(.05, .18)	.06 (.00, .13)	.11***(.05, .17)	.11***(.05, .18)	.09**(.03, .16)	.10** (.04, .17)	.10** (.04, .16)	.11***(.05, .18)
Poor childhood health	.20***(.13, .27)	.20***(.14, .27)	.19***(.13, .26)	.19***(.12, .26)	.19***(.12, .25)	.20***(.13, .27)	.19***(.12, .26)	.19***(.12, .26)
Tobacco smoking		.18***(.12, .24)						
Antipsychotic medication use			.17**(.06, .28)					
Cancer / heart attack / diabetes				.17***(.09, .24)				
Childhood socioeconomic status					-.17***(-.23, -.11)			
Childhood maltreatment						.10** (.03, .17)		
Lifetime incarceration							.17***(.07, .26)	
Early self-control difficulties								.09* (.02, .17)

Notes. All analyses were adjusted for participants' sex. Standardized regression coefficients and 95% confidence intervals are presented for all models. Analyses compare each antisocial group to the reference group "low antisocial behaviors". Model 1 presents associations adjusted for corresponding childhood measure, and serves as the comparison model. Model 2 presents associations adjusted for corresponding childhood measure and tobacco smoking. Model 3 presents associations adjusted for corresponding childhood measure, and antipsychotic medication use. Model 4 presents associations adjusted for corresponding childhood measure, and cancer/heart attack/diabetes diagnosis. Model 5 presents associations adjusted for corresponding childhood measure, and low childhood socioeconomic status. Model 6 presents associations adjusted for corresponding childhood measure and childhood maltreatment. Model 7 presents associations adjusted for corresponding childhood measure, and lifetime incarceration. Model 8 presents associations adjusted for corresponding childhood measure, and early childhood self-control difficulties. **DunedinPACE** measured within-individual decline in 19 indicators of organ-system integrity across four time points spanning two decades distilled into a single-time-point DNA-methylation blood-test using elastic-net regression. DunedinPACE can be implemented in Illumina 450 k or in EPIC array data, making it immediately available for testing in a wide range of existing datasets as a complement to existing methylation measures of aging. ***p ≤ .001, ** p ≤ .01, * p ≤ .05

Attrition Analyses

We conducted an attrition analysis using childhood SES, childhood IQ and childhood self-control to determine whether participants in the Phase 45 data collection were representative of the original cohort.

No significant differences were found between the full cohort, those deceased, those alive or those seen at Phase 45 on childhood SES.

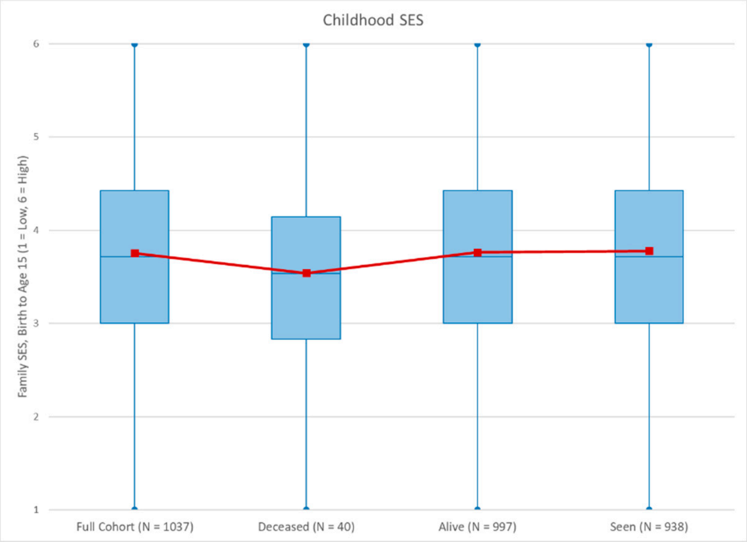

No significant differences in childhood IQ were found between the full cohort, those still alive or those seen at Phase 45. Those who were deceased by the Phase 45 data collection had significantly lower childhood IQ's than those who were still alive ($t = 2.09$, $p = 0.04$).

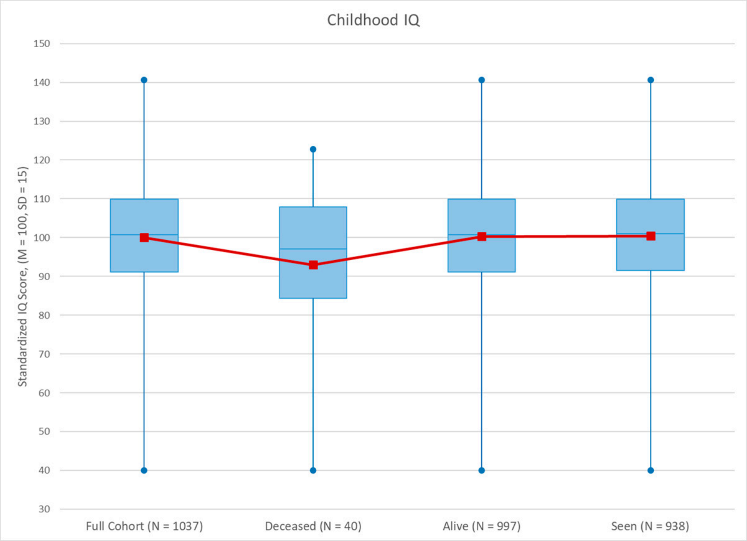

No significant differences were found between the full cohort, those deceased, those alive or those seen at Phase 45 on Childhood Low Self-Control.

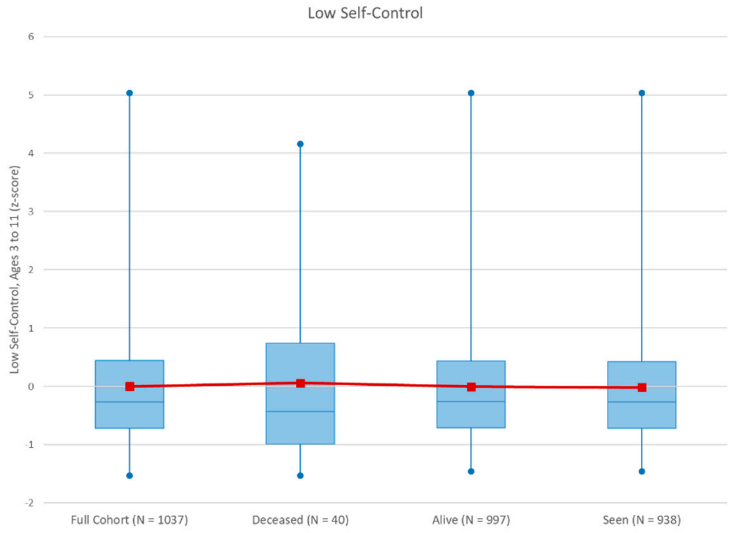

Supplementary Methods

Measuring Pace of Aging

Age 26-45 measure Pace of Aging

Our measure of the Pace of Aging is detailed elsewhere [12,33]. Our approach is guided by geroscience theory's specification that measured aging has 3 key features that distinguish it from illnesses: (1) physiological decline in one direction, (2) continuing over years of time, (3) simultaneously involving all organ systems. We operationalized this theory by modelling (1) growth curves of decline in one direction, (2) over 20 years in 4 waves of biomarker data, and (3) using biomarkers that tap the function of multiple different organ systems. We measured longitudinal changes in 19 biomarkers at ages 26, 32, 38 and 45 assessing cardiovascular, metabolic, pulmonary, renal, immune, and dental systems, totalling 69,715 data points (participants x biomarkers x assessments): Body mass index (BMI), Waist-hip ratio, Glycated hemoglobin, Leptin, Blood pressure (mean arterial pressure), Cardiorespiratory fitness (VO2Max), Total cholesterol, Triglycerides, High-density lipoprotein (HDL), Lipoprotein(a), Apolipoprotein B100/A1 ratio, Forced vital capacity ratio (FEV1/FVC), Forced expiratory volume in one second (FEV1), estimated Glomerular Filtration Rate (eGFR), Blood Urea Nitrogen (BUN), high-sensitivity C-reactive Protein (hs-CRP), White blood cell count, mean periodontal attachment loss (AL), and dental-caries-affected tooth surfaces. We then calculated each Study member's Pace of Aging in three steps. In the first step, we transformed the biomarker values to a standardized scale. For each biomarker at each wave, we standardized values according to the age-26 distribution (i.e. set to mean of 0 and a standard deviation of 1). Standardization was conducted separately for men and women. Standardized biomarker values greater than zero indicated levels that were "older" and values less than zero indicated levels "younger" than the average 26-year-old. To match, scores were reversed for VO2Max, FEV1/FVC, FEV1, eGFR, and HDL cholesterol, which are known to decline with age.

Over the 2 decades of follow-up, the biomarkers in the panel indicated a progressive deterioration of physiological integrity with advancing chronological age; i.e. their cohort mean values tended to increase (i.e., worsen) from the age-26 assessment to the age-45 assessment. In the second step, we calculated each Study member's slope for each of the 19 biomarkers—the average year-on-year change observed over the 2-decade period. Slopes were estimated using a mixed-effects growth model that regressed the biomarker's level on age. For only four of the 19 biomarkers we examined, cohort mean levels did not worsen over time as expected based on published associations with age-related chronic disease: white blood cell count and CRP levels remained stable with age; HDL cholesterol and apolipoprotein B100/A1 ratio improved with age. However, individual-difference slopes for these biomarkers did show the expected pattern of correlation with other biomarkers' slopes. For example, Study members whose apolipoprotein B100/A1 ratio increased during the follow-up period also showed increasing adiposity, declining lung function, and increasing systemic inflammation. We retained all pre-registered biomarkers in the Pace of Aging model.

In the third step, we combined information from the 19 slopes of the biomarkers to calculate each Study member's personal "Pace of Aging." Because we did not have any a priori basis for weighting differential contributions of the biomarkers to an overall Pace of Aging measure, we combined information using a unit-weighting scheme (all biomarkers were standardized to have mean=0, SD=1 based on their age-26 distributions, so slopes were denominated in comparable units). We calculated each Study member's Pace of Aging as the sum of age-dependent annual changes in biomarker Z-scores. Because the Dunedin birth cohort represents its population, its mean and distribution represent population norms. We used these norms to scale the Pace of Aging to reflect physiological change relative to the passage of time. We set the cohort mean Pace of Aging as a reference value equivalent to the physiological change expected during a single chronological year. Using this reference value, we rescaled Pace of Aging in terms of years of physiological change per chronological year (M = 1, SD = 0.29). On this scale, cohort members ranged in their Pace of Aging from 0.4 years of physiological change per chronological year (slow) to 4.3 years of physiological change per chronological year (fast).

Childhood measure of poor physical health

We measured childhood health from medical exams, anthropometry, lung function testing, and clinical interviews with parents at assessments spanning birth to age 11 years as previously described (but without including motor development) [65, 66].Children's overall health at birth, ages 3, 5, 7, 9, and 11 years was rated by two Unit staff members based on review of birth records and assessment dossiers including clinical assessments and reports of infections, diseases, injuries, hospitalizations, and other health problems collected from children's mothers during standardized interviews. Ratings were made on a five-point scale (inter-rater agreement=0.85) and reverse-coded before constructing summary measure. Body mass index was calculated from height and weight measurements taken at ages 5, 7, 9, and 11 years. In addition, tricep and subscapular skinfold thicknesses were measured at ages 7 and 9 years by trained anthropometrists (For calculation of the overall measure, tricep and subscapular skinfold thicknesses were averaged to create a single score) [67]. Systolic and diastolic blood pressure were measured at ages 7, 9, and 11 years using a London School of Hygiene and Tropical Medicine blind mercury sphygmomanometer (Cinetronics Ltd., Mildenhall, United Kingdom) [68]. Fixed expiratory volume in one second (FEV1) and the ratio of FEV1 to forced vital capacity (FVC) were measured at ages 9 and 11 using a Godart water-sealed spirometer[69] and reverse-coded before constructing summary measures. To construct a cross-age measure of childhood physical health, assessments were standardized to M=0 SD=1 within age and sex and then averaged across ages. The measure was coded so that higher scores indicated poorer childhood physical health.

<u>Measuring social hearing</u>

Age-45 measure of social hearing

Social hearing refers to listening ability in noisy environments, which requires complex processing ability. We analyzed social hearing because the ability to recognize speech in noisy environments declines with age, and deterioration in social hearing has been linked with cognitive decline[70, 71]. To measure social hearing, participants completed the Listening in

Spatialised Noise–Sentences Test (LiSN-S) (Phonak, Switzerland). All hearing tests were carried out in an acoustically attenuated room with a double door and sound-absorbing wall covering while wearing headphones. Auditory stimuli were delivered through a pair of Sennheiser 215 headphones attached to a Mini PCM2704 external sound card configured by Phonak. The LiSNS produces a three-dimensional auditory environment through the headphones via four different task conditions[31]. Target sentences spoken by a female speaker are superimposed with distractor stories (maskers). Across the four conditions, these maskers differ with respect to perceived spatial location (0o or ±90 o azimuth), and speaker identity (same or different to the target speaker). The following order of conditions was identically presented to all participants: 1) different speaker at ±90 o azimuth, 2) same speaker at ±90 o azimuth, 3) different speaker at ±0 o azimuth, and 4) same speaker at ±0 o azimuth. The masking stories were consistently presented at an intensity of 55 decibels sound pressure level (dB SPL). Participants repeated the target sentences and were scored in the software on their accuracy (words correct in each sentence). The program was adaptive, with target sentences delivered at 62 dB SPL to start, and intensity levels continuously adjusted up (if 50% of the words in the sentence correct), based on accuracy. The first few sentences (a minimum of 5) are considered practice sentences. This practice testing continues where levels were lowered in 4 dB increments, until one upward reversal in performance was recorded (i.e. the sentence score drops automatically in real time over the scored sentences, is less than 1 dB. The test condition continued until the average of the levels from positive-and negative-going reversals amounted to ≥3 (independent midpoint target level), and the standard error of these midpoints was less than 1 dB. Alternatively, the test condition continued until it reached the maximum number of 30 sentence presentations. Speech-reception thresholds were calculated as the lowest intensity at which the individual could repeat 50% of the words correctly. Our primary outcome measure was the speech-reception threshold from the low-cue condition, representing performance in the most difficult auditory environment (masker speaker same as the target speaker, and masker was presented at 0° azimuth, in the same location as the target speaker). This reflects hearing when the person is not receiving optimum auditory information.

Childhood measure of social hearing

At age 11, a speech-in-noise (SPIN) test was administered using a tape supplied by the Audiology Centre in Auckland. Six Arthur Boothroyd word lists with 10 words each[72] were presented in the following order: (1) List 1 (no noise) (2) List 2 (10 db signal/noise ratio), (3) List 3 (5 db signal/noise ratio), (4) List 4 (5 db signal/noise ratio), (5) List 5 (10 db signal/noise ratio), (6) List 6 (no noise)[36]. Words were spoken by a New Zealand male and presented at 60 dbSPL. The tape was played on a Technics stereo cassette deck model M215 attenuated through an Interacoustics AC3 Clinical Audiometer calibrated to ISO (1064) standards. Children's responses were scored phonemically as follows: 3 for a single phoneme; 7 for 2 phonemes; and 10 for the whole word giving a maximum possible score for each list of 100. A summary score was constructed for each of the three conditions (no noise, 10 dB and 5 dB signal to noise ratio), reflecting the percentage of words correctly identified (no noise condition: mean M=98.4, SD=2.4, Range 68.5-100; 10 dB condition M=90.2, SD=4.6, Range 49.5-98.5; 5db condition: M=79.3, SD=7.0, Range 29-93.5). To control for childhood hearing when testing associations with our age-45 outcome, i.e. social hearing under the most difficult auditory environment, we used the score derived from the 5 dB signal-to-noise ratio condition, measuring hearing under the most difficult auditory environment.

Measuring vision difficulties

Age-45 measure of vision difficulties

We analyzed visual contrast sensitivity, because contrast sensitivity declines with age, even after adjusting for visual acuity[73]; can be more disabling than visual acuity loss[74], and is a better predictor of mobility performance than visual acuity[75]. The ability to detect objects of different sizes at lower contrasts is expressed as a contrast sensitivity function (CSF) and determines the person's contrast detection threshold, the lowest contrast at which a pattern can be seen. Contrast sensitivity scores are linear on a logarithmic scale, and lower log CS values reflect worse contrast sensitivity. CSF testing was administered by trained visual technicians. Participants wore their glasses or contact lenses (if these were normally worn). Participants were seated one meter from the Thomson Test Chart and the Samsung 23" LCD Thin Client screen. Room lighting was set at 520 lux. Contrast sensitivity was tested with both eyes open. The Thomson Test Chart presents three letters per line and the black letters gradually fade from black to grey to white on the white background to determine the lowest level of "contrast" that the eye can detect. If only one letter on a line was correctly determined by the study member, the number of letters was recorded to determine the CSF score. However, if two letters on a line were correctly determined, the technician proceeded to the next line to determine if the study member could correctly determine any of these letters.

Childhood measure of vision difficulties

At ages 7, 9 and 11 years, participants' visual acuity was assessed using standard testing as previously described[76]. At age 7 years, visual acuity was assessed using the Sheridan Gardiner single optotype letter matching test at 6m. At age 9, and 11 years, visual acuity was measured using a 4-m logarithmic test chart. Each eye was tested separately, and the contralateral eye was occluded. The tests were performed without glasses and repeated with glasses, if they were available. A pinhole was used if the visual acuity was 6/9 or worse and glasses were not available. Testing was done in the same well-lit room at each age. Acuity testing results were converted to logMAR scores so that measures across childhood were on the same scale. On the logMAR scale, a score of 1.0 is poor vision (6/60 or 20/200 on a usual chart), a score of 0 is good vision (6/6 or 20/20 on a usual chart), and a negative score is better than 6/6 vision. For each age, we constructed a 'best-eye' visual acuity score for each study member by assigning participants the highest score they had obtained at that age. To construct a cross-age measure of childhood visual acuity assessments were standardized to M=0 SD=1 within age and then averaged across ages. This measure was reversed scored so higher scores reflect better vision.

Measuring balance difficulties

Age-45 measure of balance difficulties

We analyzed balance, because difficulties with balance increase with age[77] and are associated with reduced mobility and risk for falls[78]. Balance was measured using the Unipedal Stance Test as the maximum time achieved across three trials of the test with eyes closed[32,77,79].

Childhood measure of balance difficulties

Balance was assessed at ages 3, 7 and 9 using the balance subtests of the Bayley Motor Scales (age 3)[38] and of the Basic Motor Ability Test (ages 7 and 9)[37]. To construct a cross-age measure of childhood balance, assessments were standardized to M=0 SD=1 within age and then averaged across ages.

Measuring motor difficulties

Age-45 measure of motor difficulties

We analyzed gait speed because it is considered a geriatric vital sign and predicts multiple adverse outcomes, including frailty, disability, and mortality in older adults[80]. Gait speed (meters per second) was assessed with the 6-m-long GAITRite Electronic Walkway (CIR Systems, Inc) with 2-m acceleration and 2-m deceleration before and after the walkway, respectively, as previously described[33]. Gait speed was assessed under 3 walking conditions: usual gait speed (walk at normal pace from a standing start, measured as a mean of 2 walks) and 2 challenge paradigms, dual task gait speed (walk at normal pace while reciting alternate letters of the alphabet out loud, starting with the letter “A,” measured as a mean of 2 walks) and maximum gait speed (walk as fast as safely possible, measured as a mean of 3 walks). Gait speed was correlated across the 3 walk conditions[33]. To increase reliability and take advantage of the variation in all 3 walk conditions (usual gait and the 2 challenge paradigms), we calculated the mean of the 3 highly correlated individual walk conditions to generate our primary measure of composite gait speed.

Childhood measure of motor difficulties

Motor development was assessed at age 3 years using the Bayley Motor Scales[38], at age 5 years using the McCarthy Motor Scales[81], and at ages 7 and 9 years using the Basic Motor Ability Test[37]. To construct a cross-age measure of childhood motor development, assessments were standardized to M=0 SD=1 within age, and then averaged across ages.

Measuring cognitive difficulties

Age-45 measure of cognitive difficulties

We analyzed cognitive functioning because cognitive ability declines with age[25] and predicts survival and health in old age[83,84]. Cognitive functioning was measured by administering the Wechsler Adult Intelligence Scale-IV (WAIS-IV) [34] to the participants at age 45 years, yielding a measure of full-scale IQ, standardized to M=100, SD=15.

Childhood measure of cognitive difficulties

Participants’ cognitive functioning was individually assessed at ages 7, 9, and 11 years using the Wechsler Intelligence Scale for Children–Revised[39], yielding a measure of full-scale IQ, standardized to M=100, SD=15 at each age. To construct a cross-age measure of childhood cognitive function, assessments were averaged across ages and standardized to M=0 and SD=1.

Measuring facial age

Facial Age was based on ratings by an independent panel of eight raters of each participant's digital facial photograph. Facial Age was based on two measurements of perceived age. First, *Age Range* was assessed by an independent panel of four raters, who were presented with standardized (non-smiling) facial photographs of participants and were kept blind to their actual age. Raters used a Likert scale to categorize each participant into a 5-year age range (i.e., from 20-24 years old up to 70+ years old) (interrater reliability = 0.77). Scores for each participant were averaged across all raters. Second, *Relative Age* was assessed by a different panel of four raters, who were told that all photos were of people aged 45 years old. Raters then used a 7-item Likert scale to assign a "relative age" to each participant (1= "young looking", 7= "old looking") (interrater reliability = .79). The measure of perceived age at 45 years, Facial Age, was derived by standardizing and averaging Age Range and Relative Age scores[10,13].

<u>Measuring methylation-based pace of aging (DunedinPACE)</u>

Age-45 measure of DunedinPACE

DunedinPACE was derived by analyzing the Pace of Aging in the Dunedin Study cohort. This analysis consisted of two parts. In the first part of the analysis, Pace of Aging was derived, as previously described, from two decades of longitudinal organ-system integrity data.[31] In the second part of the analysis, we used DNA methylation data from blood collected at age 45 to derive a surrogate for the 20-year Pace of Aging measure[12,19]. Specifically, elastic-net-regression was used to develop a DNA methylation algorithm to predict the 20-year Pace of Aging. Analysis included the subset of probes included on both the Illumina 450K and EPIC arrays which we previously determined to have acceptable test-retest reliability[85]. The resulting algorithm included 173 CpG sites. The DunedinPACE algorithm is available to the research community on GitHub as an R package[19].

Covariates

<u>Measuring health problems in adulthood</u>

Tobacco smoking was coded as whether participants had reported daily smoking at any assessment up to age 45 years (478 participants [51.6% of cohort study members]).

Antipsychotic medication use at age 45 years was assessed in standardized interviews about their medications, and participants brought medications on the assessment day, which were evaluated by a pharmacist. Antipsychotics were used by 18 cohort study members (1.9%).

Cancer or heart attack by age 45 years was assessed by standardized interviews that ascertained whether participants had been told by a health professional that they had any of several diseases. Diabetes was assessed based on participants' blood levels of glycated hemoglobin[12]. In line with clinical diagnostic criteria, a cutoff of 48 mmol/mol was used. Cancer, heart attacks, or diabetes by age 45 years affected 58 participants (6.2% of cohort study members).

<u>Measuring adverse experiences</u>

Childhood maltreatment[42] includes evidence of (1) maternal rejection assessed at age 3 years by observational ratings of mothers' interaction with the study children, (2) harsh discipline assessed at ages 7 and 9 years by parental report of disciplinary behaviors, (3) 2 or more changes in the child's primary caregiver, and (4) physical abuse and (5) sexual abuse reported by study members once they reached adulthood. For each child, our cumulative index counts the number of maltreatment indicators during the first decade of life; 64.2% of cohort study members experienced no maltreatment, 26.6% experienced 1 indicator of maltreatment ("probable" maltreatment), and 9.2% experienced 2 or more indicators of maltreatment ("definite" maltreatment).

Childhood socioeconomic status of participants' families was measured using a 6-point scale that assessed parents' occupational statuses, defined based on average income and educational levels derived from the New Zealand Census[41].

Lifetime incarceration was assessed via self-report at ages 32, 38 and 45 years. At age 32, participants were asked, "In your life, have you ever spent any time in jail or prison?" At phase 38 and 45, this was updated with months of incarceration between phases. These were combined to create lifetime months of incarceration. A total of 39 cohort study members (4.0%) were identified as having spent time at least one month in jail or prison.

<u>Measuring early childhood self-control</u>

Early childhood self-control was assessed at ages 3 and 5 years. Children's self-control was rated based on observation by trained research assistants during 90-minutes data collection sessions at the research unit[44]. Each study child participated in a testing session involving cognitive and motor tasks. The children were tested by examiners who had no knowledge of their behavioral history. Following the testing, each examiner rated the child's lack of control in the testing session[43].

<u>Measuring health knowledge</u>

Study members' practical *health knowledge* at age 45 was indexed by two scales:

Multiple-choice assessment. Participants were administered a six-item multiple-choice assessment of their understanding of different health principles, including those related to medical knowledge, prevention, aging, physical disease, sun exposure, and sleep. The number of correct responses was summed to create a scale (range=0-6, α=0.41) [26].

Open-ended interview. Participants were interviewed about their understanding of different health principles, with an open-ended response format: "What are some of the reasons you should know your family history of illness?"; "If you are sick and the doctor gives you an antibiotic, what are some of the reasons why you should finish all the pills?"; "What are some of the reasons it is important to get your blood pressure checked?"; "What are some of the reasons you should wear sunglasses when out on a sunny day?" "What are some of the reasons you should get regular sleep?"; and "What are some of the reasons people tend to gain weight as they

get older?” Using standardized scoring procedures, four trained raters (two raters per interview) coded responses on a scale from 0 to 2, with 0 indicating no understanding of the health principle, 1 indicating moderate understanding, and 2 indicating good understanding (interrater reliability=0.94). For instance, in response to the question “What are some of the reasons you should wear sunglasses when out on a sunny day?”, the following responses were coded as 0, 1, and 2, respectively: “To prevent squinting,” “To prevent eye damage,” and “To protect your eyes from UV rays.” Scale scores were computed by summing across the items and then averaging across raters (range=1-12) [26].

The multiple-choice and open-ended scales were correlated ($r=0.39$, $p<.0001$). The Practical Health Knowledge measure was computed by standardizing ($M=0$, $SD=1$) and averaging the multiple-choice and open-ended scales.
